# Patient-reported outcomes during repetitive oxaliplatin-based pressurized intraperitoneal aerosol chemotherapy for isolated unresectable colorectal peritoneal metastases in a multicenter, single-arm, phase 2 trial (CRC-PIPAC)

**DOI:** 10.1007/s00464-021-08802-6

**Published:** 2021-11-10

**Authors:** Robin J. Lurvink, Koen P. Rovers, Emma C. E. Wassenaar, Checca Bakkers, Jacobus W. A. Burger, Geert-Jan M. Creemers, Maartje Los, Floortje Mols, Marinus J. Wiezer, Simon W. Nienhuijs, Djamila Boerma, Ignace H. J. T. de Hingh

**Affiliations:** 1 Department of Surgery, Catharina Cancer Institute, PO Box 1350, 5602 ZA Eindhoven, The Netherlands; 2 Department of Research and Development, Netherlands Comprehensive Cancer Organization, Utrecht, The Netherlands; 3 Department of Surgery, St. Antonius Hospital, PO Box 2500, 3430 EM Nieuwegein, The Netherlands; 4 Department of Medical Oncology, Catharina Cancer Institute, PO Box 1350, 5602 ZA Eindhoven, The Netherlands; 5 Department of Medical Oncology, St. Antonius Hospital, PO Box 2500, 3430 EM Nieuwegein, The Netherlands; 6 Center of Research on Psychology in Somatic Disorders, Department of Medical and Clinical Psychology, Tilburg University, PO Box 90153, 5000 LE Tilburg, The Netherlands; 7 GROW - School for Oncology and Developmental Biology, Maastricht University, PO Box 616, 6200 MD Maastricht, The Netherlands

**Keywords:** Colorectal neoplasms, Peritoneal neoplasms, Antineoplastic agents, Oxaliplatin, Patient-reported outcome measures, Quality of life

## Abstract

**Background:**

CRC-PIPAC prospectively assessed repetitive oxaliplatin-based pressurized intraperitoneal aerosol chemotherapy (PIPAC-OX) as a palliative monotherapy (i.e., without concomitant systemic therapy in between subsequent procedures) for unresectable colorectal peritoneal metastases (CPM). The present study explored patient-reported outcomes (PROs) during trial treatment.

**Methods:**

In this single-arm phase 2 trial in two tertiary centers, patients with isolated unresectable CPM received 6-weekly PIPAC-OX (92 mg/m^2^). PROs (calculated from EQ-5D-5L, and EORTC QLQ-C30 and QLQ-CR29) were compared between baseline and 1 and 4 weeks after the first three procedures using linear mixed modeling with determination of clinical relevance (Cohen’s *D* ≥ 0.50) of statistically significant differences.

**Results:**

Twenty patients underwent 59 procedures (median 3 [range 1–6]). Several PROs solely worsened 1 week after the first procedure (index value − 0.10, *p* < 0.001; physical functioning − 20, *p* < 0.001; role functioning − 27, *p* < 0.001; social functioning − 18, *p* < 0.001; C30 summary score − 16, *p* < 0.001; appetite loss + 15, *p* = 0.007; diarrhea + 15, *p* = 0.002; urinary frequency + 13, *p* = 0.004; flatulence + 13, *p* = 0.001). These PROs returned to baseline at subsequent time points. Other PROs worsened 1 week after the first procedure (fatigue + 23, *p* < 0.001; pain + 29, *p* < 0.001; abdominal pain + 32, *p* < 0.001), second procedure (fatigue + 20, *p* < 0.001; pain + 21, *p* < 0.001; abdominal pain + 20, *p* = 0.002), and third procedure (pain + 22, *p* < 0.001; abdominal pain + 22, *p* = 0.002). Except for appetite loss, all changes were clinically relevant. All analyzed PROs returned to baseline 4 weeks after the third procedure.

**Conclusions:**

Patients receiving repetitive PIPAC-OX monotherapy for unresectable CPM had clinically relevant but reversible worsening of several PROs, mainly 1 week after the first procedure.

**Trial registration:**

Clinicaltrials.gov: NCT03246321; Netherlands trial register: NL6426.

**Supplementary Information:**

The online version contains supplementary material available at 10.1007/s00464-021-08802-6.

The peritoneum is a common and often lethal metastatic site of colorectal cancer [1, 2]. The majority of patients with colorectal peritoneal metastases (CPM) are treated with palliative intent [3, 4]. Theoretically, intraperitoneal chemotherapy could be an interesting palliative treatment option in patients with isolated peritoneal metastases, as it may achieve high locoregional efficacy with low systemic toxicity [5]. However, its use appears to be limited due to an inhomogeneous intraperitoneal distribution, dose-limiting local toxicity, and poor tumor penetration [6, 7]. To overcome these limitations, a laparoscopic method for the repetitive delivery of low-dose intraperitoneal chemotherapy as a pressurized aerosol (i.e., pressurized intraperitoneal aerosol chemotherapy [PIPAC]) has been developed. PIPAC claims to result in enhanced tumor penetration, homogeneous intraperitoneal distribution, and low systemic toxicity [8–11]. The intriguing concept and promising preliminary results have led to the adoption of PIPAC as a palliative treatment option for isolated unresectable CPM in a rapidly increasing number of hospitals worldwide [12]. In these hospitals, these patients regularly receive oxaliplatin-based PIPAC (PIPAC-OX) with or without concomitant palliative systemic therapy in a dose of 90–92 mg/m^2^ every 4 to 8 weeks [13]. Despite its increasing use, repetitive PIPAC-OX has never been prospectively investigated as a palliative monotherapy (i.e., without palliative systemic therapy in between subsequent procedures) for isolated unresectable CPM in clinical trials. As a first step to address this evidence gap, the CRC-PIPAC trial primarily aimed to assess the feasibility, safety, preliminary efficacy, survival outcomes, and patient-reported outcomes (PROs) of repetitive PIPAC-OX monotherapy for isolated unresectable CPM [14, 15]. The aim of the present study was to explore PROs during trial treatment.

## Materials and methods

### Trial design

CRC-PIPAC was a single-arm phase 2 trial conducted in two Dutch tertiary centers for the surgical treatment of CPM. The trial was approved by a central ethics committee (MEC-U, Nieuwegein, Netherlands, R17.038) and the institutional review boards of both trial centers. The trial is registered (Clinicaltrials.gov: NCT03246321), and the protocol has been previously published [14].

### Patients

The protocol includes a detailed description of the eligibility criteria [14]. Briefly, eligible patients were adults with a World Health Organization performance status of 0–1, pathologically proven isolated unresectable peritoneal metastases of a colorectal or appendiceal carcinoma (or high-grade appendiceal mucinous neoplasm), adequate organ functions, no symptoms of gastrointestinal obstruction, no contraindications for laparoscopy or the planned chemotherapy, and no previous PIPAC, in any line of palliative treatment. Patients were informed about the potential consequences of discontinuing or postponing standard palliative treatment, were discussed in a multidisciplinary team prior to enrollment, and gave written informed consent.

### Procedures

#### PIPAC-OX

The protocol comprises a detailed description of the procedure [14], which is based on internationally used protocols [13]. Patients underwent 6-weekly PIPAC-OX (92 mg/m^2^, maximum 184 mg) with a simultaneous intravenous bolus 5-fluorouracil (400 mg/m^2^) and leucovorin (20 mg/m^2^) [16, 17] and electrostatic aerosol precipitation (i.e., ePIPAC-OX) [18, 19]. Electrostatic precipitation was started directly after complete injection of the aerosol, after which the total procedure was maintained for 25 min. If possible, patients were discharged on the first postoperative day. No concomitant palliative systemic therapy was given in between subsequent procedures (i.e., ePIPAC-OX monotherapy).

#### Evaluations

Four weeks after each procedure, patients were clinically, biochemically, and radiologically evaluated. Trial treatment was stopped in case of radiological progression according to the response evaluation criteria in solid tumors (RECIST) [20]. In case of RECIST non-evaluable or stable disease (or response), the decision to continue or stop trial treatment was made by shared decision based on previous treatment, remaining treatment options, clinical parameters (e.g., toxicity, symptoms), biochemical parameters (e.g., tumor markers), macroscopic parameters (e.g., ascites volume), and secondary radiological parameters (e.g., radiological peritoneal cancer index). If trial treatment was stopped, patients received off-protocol palliative treatment.

### PROs

Patients were asked to complete three questionnaires (EuroQoL EQ-5D-5L [21], EORTC QLQ-C30 [22], and EORTC QLQ-CR29 [23] at baseline and 1 and 4 weeks after each procedure. As the trial’s aim was to assess PROs during trial treatment, patients were not asked to complete questionnaires after discontinuation of trial treatment (e.g., due to disease progression) or during follow-up. At patient’s preference, questionnaires were sent on paper or electronically using certified software (Research Manager, Deventer, the Netherlands). Table 1 presents the PRO categories of each questionnaire. Scores for each category were calculated according to the manuals of EuroQol and EORTC [24–26]. Scores range from 0 to 100 except for the index value of EQ-5D-5L, which ranges from − 0.329 to 1.00 according to the Dutch value set [27]. In general, PRO categories can be divided in function scales (with lower scores indicating worse functioning) and symptom scales (with higher scores indicating worse symptoms) (Table 1).

**Table 1:** PROs of each questionnaire

Questionnaire	Function scales^a^	Symptom scales^b^
EQ-5D-5L	• Visual analog scale • Index value	
EORTC QLQ-C30	• Global health status • Physical functioning • Role functioning • Emotional functioning • Cognitive functioning • Social functioning • C30 summary score	• Fatigue • Nausea/vomiting • Pain • Dyspnea • Insomnia • Appetite loss • Constipation • Diarrhea • Financial difficulties
EORTC QLQ-CR29	• Anxiety • Weight • Body image • Sexual interest (males) • Sexual interest (females)	• Urinary frequency • Urinary incontinence • Dysuria • Abdominal pain • Buttock pain • Bloating • Blood/mucus in stool • Dry mouth • Hair loss • Taste • Flatulence • Fecal incontinence • Sore skin • Stool frequency • Embarrassment • Stoma care problems • Impotence (males) • Dyspareunia (females)

^a^Lower scores indicate worse functioning

^b^Higher scores indicate worse symptoms

### Statistical analysis

Since no data were available to guide a sample size calculation, the investigators and the ethics committee agreed upon a sample size of 20 patients undergoing an estimated number of 60 procedures as sufficient numbers to explore the safety and feasibility of the intervention in the CRC-PIPAC trial. As PRO assessment of the present study was explorative, no a priori hypothesis for PRO analyses was formulated. Consequently, the scores of all PRO categories up to 4 weeks after the third procedure were included in the analyses. Analyses were performed two sided using IBM SPSS Statistics (version 25.0, Armonk, NY, United States). To account for multiple testing, Bonferroni corrections were applied for each PRO category. Hence, *p* < 0.0083 was considered statistically significant (i.e., *α* = 0.05 divided by 6 comparisons per PRO category). For each PRO category, changes in scores between baseline and subsequent time points were presented as a mean difference (MD) and analyzed using linear mixed modeling. Pairwise deletion was used in case of missing values. All PRO categories with a statistically significant difference in scores between baseline and at least one subsequent time point were presented. For all PRO categories, Cohen’s d (CD) effect sizes were calculated to determine the clinical relevance of each statistically significant difference, with a CD of ≥ 0.5 being considered clinically relevant [28]. Furthermore, patient-based clinical thresholds were used to determine whether deteriorations or improvements of PROs were major, moderate, or minor (EORTC QLQ-C30 [29], EORTC QLQ-CR29) [30] and to determine whether a change in a PRO exceeded a minimally important difference (EuroQol EQ-5D-5L) [31]. Since mean scores were used to present changes over time and determine effect sizes, all scores were presented as a mean (standard deviation) regardless of distribution.

## Results

**Fig. 1:**
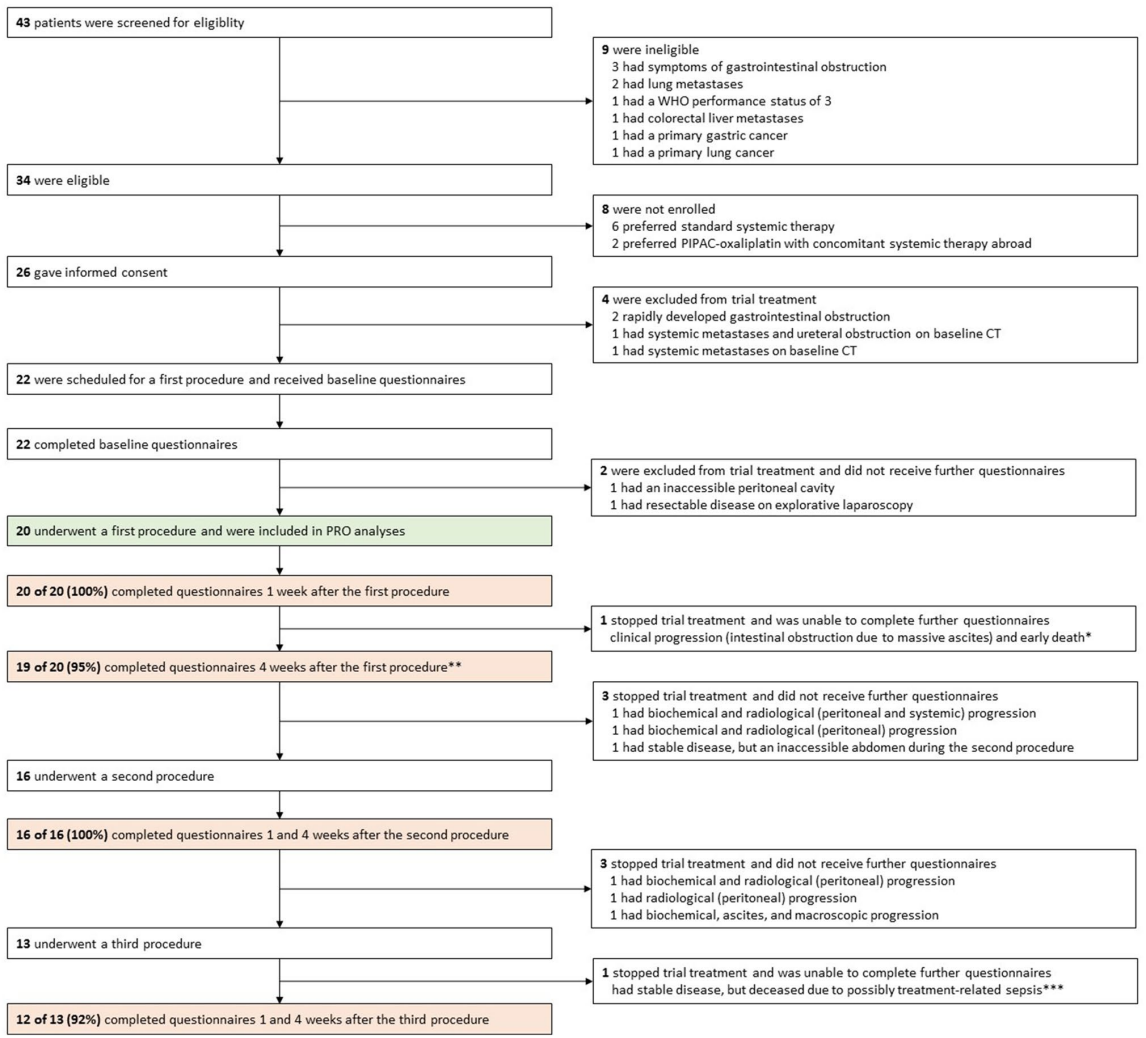
Patient pathway and questionnaire response rates (including reasons for non-response) at each time point. *WHO* world health organization; *deceased 2 weeks postoperatively; **one patient completed EQ-5D-5L and EORTC QLQ-CR29, but accidentally forgot to fill in most questions of EORTC QLQ-CR30; ***deceased five days postoperatively

Between October 10, 2017, and September 24, 2018, 43 patients were screened for eligibility, of whom 20 were included in the PRO analyses (Fig. 1). Baseline characteristics are presented in Table 2. Between October 30, 2017, and April 24, 2019, these 20 patients underwent 59 (median 3 [range 1–6]) procedures. Figure 1 presents the patient pathway and questionnaire response rates at each time point, including reasons for non-response and discontinuation of trial treatment.

**Table 2:** Baseline characteristics

Sex	20
Male	12 (60%)
Female	8 (40%)
Years of age at enrollment, median (range)	64 (41–78)
WHO performance status	**20**
0	8 (40%)
1	10 (50%)
≥ 2^a^	2 (10%)^a^
Primary tumor location	**20**
Right colon	7 (35%)
Left colon	6 (30%)
Appendix	7 (35%)
Histology: primary tumor in colon	**13**
Adenocarcinoma	4 (31%)
Mucinous adenocarcinoma	5 (38%)
Signet ring cell carcinoma	4 (31%)
Histology: primary tumor in appendix	**7**
Mucinous adenocarcinoma	1 (14%)
Signet ring cell carcinoma	4 (57%)
Low-grade appendiceal mucinous neoplasm^b^	2 (29%)^b^
Primary tumor resection status	**20**
Resected	6 (30%)
In situ, but diverted or bypassed	5 (25%)
In situ and not diverted or bypassed	9 (45%)
Onset of peritoneal metastases	**20**
Synchronous	15 (75%)
Metachronous	5 (25%)
Months between diagnosis of peritoneal metastases and enrollment, median (range)	4 (1–32)
Previous systemic therapy	**20**
Yes, with oxaliplatin	11 (55%)
Yes, without oxaliplatin	1 (5%)
No	8 (40%)
Previous systemic therapy: synchronous peritoneal metastases	**15**
None^c^	6 (40%)^c^
One line of palliative systemic therapy	8 (53%)
Multiple lines of palliative systemic therapy	1 (7%)
Previous systemic therapy: metachronous peritoneal metastases	**5**
None^c^	2 (40%)^c^
Adjuvant systemic therapy only^c^	2 (40%)^c^
Adjuvant systemic therapy and multiple lines of palliative systemic therapy	1 (20%)
Latest response to palliative treatment	**11**^**d**^
Stable disease	8 (73%)
Progression	3 (27%)^d^
Previous laparotomies	**20**
None	13 (65%)
One	6 (30%)
Multiple	1 (5%)
Ascites	**20**
Yes, ≥ 50 ml	13 (65%)
Yes, < 50 ml	3 (15%)
No	4 (20%)
Ascites volume (ml), median (range)^e^	260 (60–6000)^e^
Radiological peritoneal cancer index at baseline radiology, median (range)	31 (11–39)
Macroscopic peritoneal cancer index during first laparoscopy, median (range)	29 (17–39)

*WHO* world health organization

^a^Both deteriorated between enrollment (WHO 1) and the first procedure (one WHO 2, one WHO 3) due to increasing ascites

^b^Pre-trial biopsies classified as high-grade appendiceal mucinous neoplasm, but biopsies during the trial revealed low-grade appendiceal mucinous neoplasm

^c^Either refused—or preferred enrollment rather than starting with—first- or second-line palliative systemic therapy

^d^One had a wait-and-see strategy

^e^In those with ≥ 50 ml

Table 3 presents the mean scores of all PRO categories at each time point. Online Appendix A presents the linear mixed modeling analyses of the 12 PRO categories with a statistically significant difference*.* The scores of all other 29 PRO categories did not significantly change during trial treatment (linear mixed modeling analyses presented in Online Appendix B).

**Table 3:** Mean scores with standard deviations of all PROs at each time point

EuroQoL EQ-5D-5L							
PRO	Baseline	1 week after 1st procedure	4 weeks after 1st procedure	1 week after 2nd procedure	4 weeks after 2nd procedure	1 week after 3rd procedure	4 weeks after 3rd procedure
Visual analog scale	62 ± 28	54 ± 29	64 ± 24	64 ± 21	59 ± 32	66 ± 23	63 ± 30
Index value	0.84 ± 0.11	0.74 ± 0.15	0.82 ± 0.14	0.81 ± 0.12	0.83 ± 0.14	0.80 ± 0.18	0.85 ± 0.14

EORTC QLQ-C30							
PRO	Baseline	1 week after 1st procedure	4 weeks after 1st procedure	1 week after 2nd procedure	4 weeks after 2nd procedure	1 week after 3rd procedure	4 weeks after 3rd procedure
Global health status	65 ± 29	57 ± 21	67 ± 25	60 ± 25	65 ± 24	63 ± 22	67 ± 24
Physical functioning	87 ± 15	67 ± 23	81 ± 20	76 ± 18	81 ± 20	78 ± 19	86 ± 20
Role functioning	69 ± 30	42 ± 32	69 ± 28	53 ± 31	64 ± 39	56 ± 30	76 ± 29
Emotional functioning	79 ± 23	78 ± 23	81 ± 18	74 ± 20	78 ± 23	78 ± 22	76 ± 23
Cognitive functioning	88 ± 17	82 ± 17	91 ± 12	87 ± 16	96 ± 10	90 ± 13	90 ± 17
Social functioning	80 ± 27	62 ± 24	79 ± 23	79 ± 25	81 ± 25	76 ± 32	85 ± 30
Fatigue	30 ± 23	53 ± 24	37 ± 25	50 ± 25	35 ± 30	43 ± 28	33 ± 28
Nausea/vomiting	13 ± 25	19 ± 27	8 ± 13	11 ± 19	7 ± 12	14 ± 17	6 ± 15
Pain	21 ± 20	50 ± 19	29 ± 18	42 ± 24	29 ± 27	43 ± 26	25 ± 23
Dyspnea	12 ± 20	13 ± 20	9 ± 19	15 ± 24	6 ± 13	8 ± 21	6 ± 19
Insomnia	17 ± 23	23 ± 31	24 ± 22	31 ± 28	19 ± 24	17 ± 17	14 ± 22
Appetite loss	25 ± 37	40 ± 32	24 ± 36	37 ± 34	27 ± 33	39 ± 31	25 ± 32
Constipation	8 ± 15	22 ± 27	13 ± 26	12 ± 21	6 ± 13	11 ± 22	3 ± 10
Diarrhea	12 ± 20	27 ± 26	19 ± 21	6 ± 13	10 ± 16	17 ± 27	14 ± 17
Financial difficulties	3 ± 15	10 ± 27	2 ± 8	2 ± 8	0 ± 0	3 ± 10	3 ± 10
C30 summary score	82 ± 15	66 ± 15	80 ± 14	75 ± 16	81 ± 16	76 ± 16	84 ± 18

EORTC QLQ-CR29							
PRO	Baseline	1 week after 1st procedure	4 weeks after 1st procedure	1 week after 2nd procedure	4 weeks after 2nd procedure	1 week after 3rd procedure	4 weeks after 3rd procedure
Urinary frequency	12 ± 17	25 ± 18	24 ± 20	26 ± 18	21 ± 22	26 ± 19	19 ± 16
Urinary incontinence	2 ± 7	0 ± 0	4 ± 11	4 ± 11	4 ± 12	3 ± 10	0 ± 0
Dysuria	3 ± 10	7 ± 14	7 ± 18	6 ± 13	10 ± 26	6 ± 13	6 ± 13
Abdominal pain	20 ± 17	52 ± 23	39 ± 25	40 ± 28	35 ± 33	42 ± 25	22 ± 26
Buttock pain	7 ± 14	5 ± 16	11 ± 19	6 ± 13	6 ± 13	8 ± 15	3 ± 10
Bloating	28 ± 27	35 ± 23	23 ± 25	33 ± 30	29 ± 32	28 ± 31	22 ± 30
Blood/mucus in stool	0 ± 0	3 ± 9	3 ± 6	2 ± 6	3 ± 7	1 ± 5	0 ± 0
Dry mouth	18 ± 23	27 ± 32	24 ± 25	25 ± 23	19 ± 21	22 ± 22	17 ± 22
Hair loss	3 ± 10	5 ± 12	5 ± 12	4 ± 11	8 ± 19	8 ± 21	11 ± 30
Taste	18 ± 30	22 ± 31	16 ± 23	27 ± 30	23 ± 26	19 ± 26	14 ± 22
Flatulence	17 ± 20	30 ± 26	21 ± 23	15 ± 17	8 ± 15	14 ± 17	14 ± 17
Fecal incontinence	5 ± 16	5 ± 12	4 ± 11	2 ± 8	2 ± 8	3 ± 10	0 ± 0
Sore skin	7 ± 14	10 ± 19	11 ± 19	2 ± 8	4 ± 11	6 ± 13	6 ± 19
Stool frequency	8 ± 11	15 ± 23	5 ± 8	7 ± 10	6 ± 10	7 ± 15	7 ± 11
Embarrassment	10 ± 19	10 ± 19	7 ± 14	10 ± 16	4 ± 11	3 ± 10	3 ± 10
Stoma care problems	8 ± 17	8 ± 17	0 ± 0	17 ± 24	33 ± 47	17 ± 24	17 ± 24
Impotence (males)	3 ± 10	15 ± 23	12 ± 17	8 ± 15	8 ± 15	13 ± 17	4 ± 12
Dyspareunia (females)	4 ± 12	0 ± 0	0 ± 0	0 ± 0	10 ± 16	11 ± 19	17 ± 19
Anxiety	53 ± 29	62 ± 33	60 ± 33	56 ± 32	60 ± 35	56 ± 30	61 ± 28
Weight	80 ± 33	77 ± 33	84 ± 23	83 ± 24	75 ± 31	75 ± 35	78 ± 36
Body image	87 ± 17	83 ± 23	90 ± 14	84 ± 22	85 ± 21	82 ± 25	81 ± 23
Sexual interest (males)	31 ± 30	22 ± 26	25 ± 29	22 ± 24	19 ± 24	21 ± 25	25 ± 30
Sexual interest (females)	8 ± 15	4 ± 12	10 ± 16	5 ± 13	10 ± 16	0 ± 0	8 ± 17

*PRO* patient-reported outcome

### Changing function scales

#### Index value

**Fig. 2:**
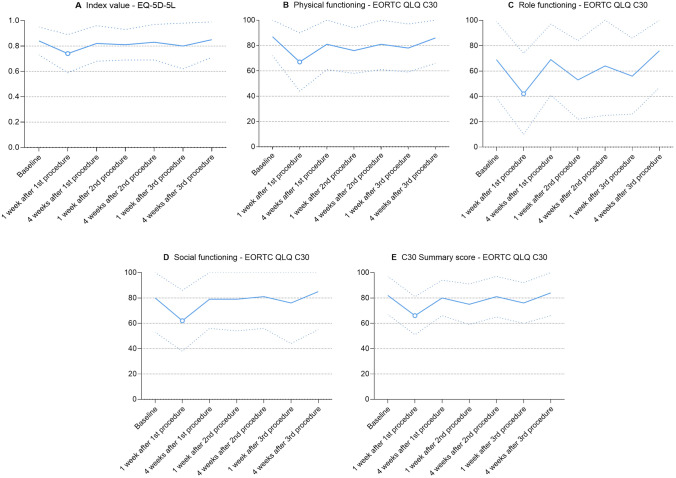
All function scales with a statistically significant difference in scores between baseline and at least one subsequent time point. Blue lines represent mean scores; dotted blue lines represent standard deviations; hollow dots represent statistically significant and clinically relevant differences compared to baseline

Compared to baseline, index value worsened 1 week after the first procedure (MD − 0.10 [95% confidence interval: − 0.16 to − 0.05], *p* < 0.001, CD 0.76, exceeds minimally important difference) and returned to baseline at subsequent time points (Fig. 2A).

#### Physical functioning

Compared to baseline, physical functioning worsened 1 week after the first procedure (MD − 20 [95% CI − 27 to − 12], *p* < 0.001, CD 1.03, major deterioration) and returned to baseline at subsequent time points (Fig. 2B).

#### Role functioning

Compared to baseline, role functioning worsened 1 week after the first procedure (MD − 27 [95% CI − 39 to − 15], *p* < 0.001, CD 0.87, major deterioration) and returned to baseline at subsequent time points (Fig. 2C).

#### Social functioning

Compared to baseline, social functioning worsened 1 week after the first procedure (MD − 18 [95% CI − 28 to − 8], *p* < 0.001, CD 0.71, moderate deterioration) and returned to baseline at subsequent time points (Fig. 2D).

#### C30 summary score

Compared to baseline, C30 summary score worsened 1 week after the first procedure (MD − 16 [95% CI − 20 to − 9], *p* < 0.001, CD 1.07, moderate deterioration) and returned to baseline at subsequent time points (Fig. 2E).

### Changing symptom scales

#### Fatigue

**Fig. 3:**
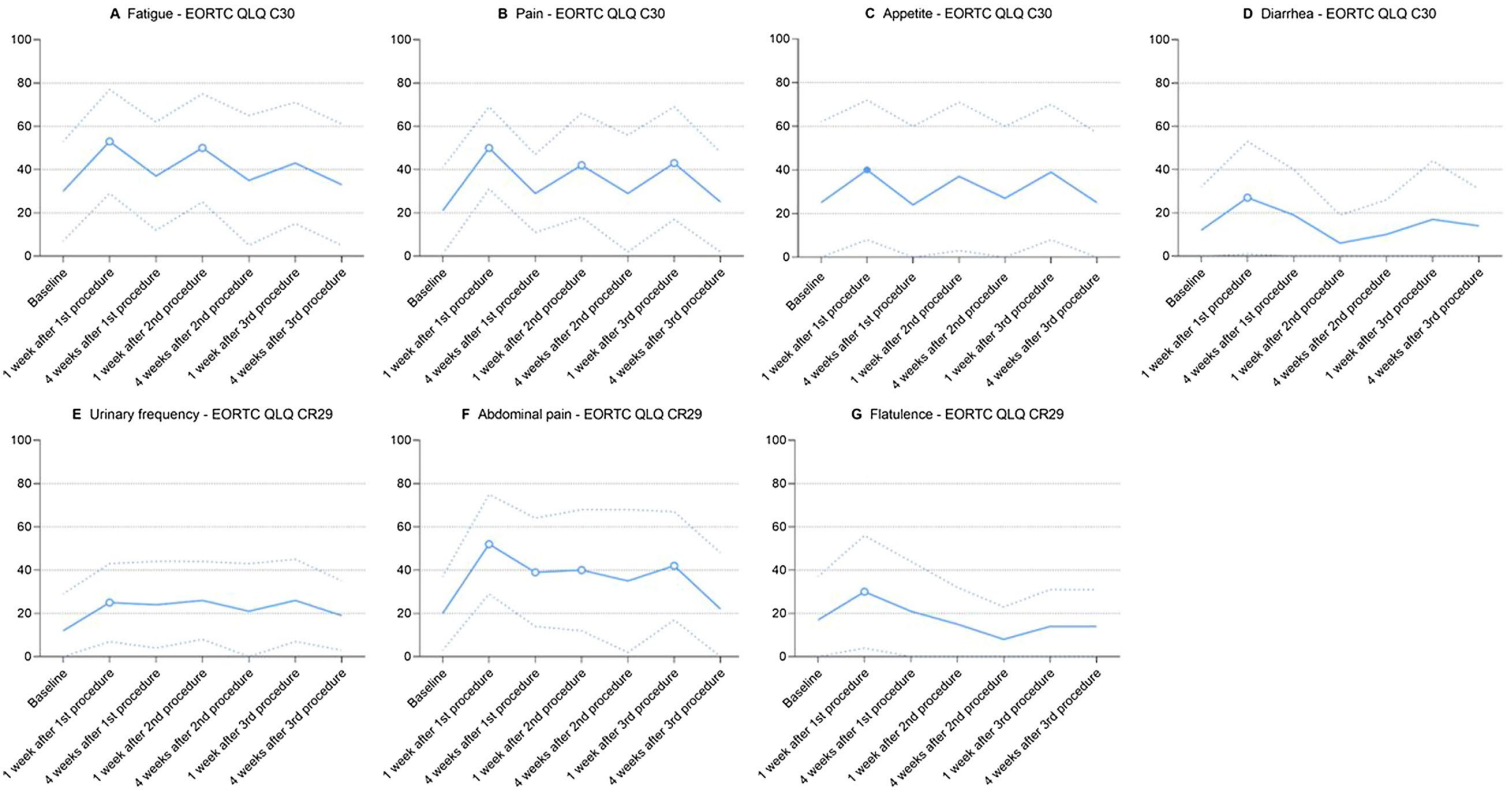
All symptom scales with a statistically significant difference in scores between baseline and at least one subsequent time point. Blue lines represent mean scores; dotted blue lines represent standard deviations; hollow dots represent statistically significant and clinically relevant differences compared to baseline; filled dots represent statistically significant but clinically irrelevant differences compared to baseline

Compared to baseline, fatigue worsened 1 week after the first procedure (MD + 23 [95% CI 14–33], *p* < 0.001, CD 0.98, major deterioration), returned to baseline 4 weeks after the first procedure (*p* = 0.13), worsened 1 week after the second procedure (MD + 20 [95% CI 9–30], *p* < 0.001, CD 0.83, major deterioration), and returned to baseline at subsequent time points (Fig. 3A).

#### Pain

Compared to baseline, pain was worse 1 week after the first procedure (MD + 29 [95% CI 19–40], *p* < 0.001, CD 1.49, major deterioration), second procedure (MD + 21 [95% CI 10–33], *p* < 0.001, CD 0.95, major deterioration), and third procedure (MD + 22, [95% CI 11–36], *p* < 0.001, CD 0.95, major deterioration), and was comparable to baseline 4 weeks after each procedure (Fig. 3B).

#### Appetite loss

Compared to baseline, appetite loss worsened 1 week after the first procedure (MD + 15 [95% CI 4–26], *p* = 0.007, CD 0.43, moderate deterioration) and returned to baseline at subsequent time points (Fig. 3C).

#### Diarrhea

Compared to baseline, diarrhea worsened 1 week after the first procedure (MD + 15 [95% CI 5–25], *p* = 0.002, CD 0.65, minor deterioration) and returned to baseline at subsequent time points (Fig. 3D).

#### Urinary frequency

Compared to baseline, urinary frequency worsened 1 week after the first procedure (MD + 13 [95% CI 4–22], *p* = 0.004, CD 0.74, moderate deterioration) and returned to baseline at subsequent time points (Fig. 3E).

#### Abdominal pain

Compared to baseline, abdominal pain was worse 1 week after the first procedure (MD + 32 [95% CI 20–43], *p* < 0.001, CD 1.58, major deterioration), 4 weeks after the first procedure (MD + 19 [95% CI 7–31], *p* = 0.003, CD 0.89, moderate deterioration), 1 week after the second procedure (MD + 20 [95% CI 7–33], *p* = 0.002, CD 0.86, moderate deterioration), and 1 week after the third procedure (MD + 22 [95% CI 9–36], *p* = 0.002, CD 1.03, major deterioration), and was comparable to baseline 4 weeks after the second and third procedures (Fig. 3F).

#### Flatulence

Compared to baseline, flatulence worsened 1 week after the first procedure (MD + 13 [95% CI 6–21], *p* = 0.001, CD 0.56, moderate deterioration) and returned to baseline at subsequent time points (Fig. 3G).

## Discussion

The present study showed that patients with isolated unresectable CPM receiving repetitive ePIPAC-OX monotherapy had worsening of several general (index value, physical functioning, role functioning, social functioning, C30 summary score, fatigue) and more specific (pain, appetite loss, diarrhea, urinary frequency, abdominal pain, flatulence) PROs during trial treatment. The majority of these PROs worsened 1 week after the first procedure and returned to baseline at all subsequent time points. However, fatigue also worsened 1 week after the second procedure, and (abdominal) pain worsened 1 week after all three procedures. All worsening PROs (except for appetite loss) were clinically relevant, but all eventually returned to baseline scores 4 weeks after the third procedure. Despite these changes in several PROs, it should also be noted that all other analyzed PROs did not change during trial treatment. This is a promising finding in this vulnerable study population (high disease burden, eight patients with signet ring cell differentiation) who generally have a poor prognosis with potentially rapidly deteriorating quality of life. 

Thirteen other studies assessed PROs in patients undergoing PIPAC for peritoneal metastases [32–44]. However, six of these specifically focused on PIPAC-cisplatin-doxorubicin for non-colorectal primaries (ovarian cancer [32, 33], gastric cancer [34, 35], peritoneal mesothelioma [36], and endometrial and breast cancer [37]. Six other studies [38–43], all focusing on PIPAC with various drugs for various primaries (including oxaliplatin for colorectal cancer), did not report specific outcomes for (e)PIPAC-OX for CPM. Only one of thirteen studies specifically assessed PROs of PIPAC-OX for CPM [44]. However, this study did not report separate PROs of PIPAC-OX monotherapy and PIPAC-OX with concomitant systemic therapy. Altogether, the results of the present study could not be meaningfully compared with the existing literature. Hence, the present study provides detailed insights into PROs during repetitive (e)PIPAC-OX monotherapy for isolated unresectable CPM. Results of the present study may be used to inform patients and physicians about the burden and side effects of ePIPAC-OX in this setting. Several arguments may indicate that the worsening of PROs was most likely related to ePIPAC-OX rather than concomitant treatments or disease progression. First, none of the patients in the present study received concomitant systemic therapy. Second, patients with disease progression did not receive further questionnaires. Third, in all worsening PROs, this worsening was seen at 1 week postoperatively and not at 4 weeks postoperatively. The possibility of treatment-related worsening of PROs should be taken into account by physicians when proposing (e)PIPAC-OX in the palliative setting.

Nevertheless, other treatments for CPM may affect PROs as well. Two studies reported a gradual deterioration of several PROs during treatment with systemic chemotherapy, being the global health status, physical functioning, social functioning, emotional functioning, fatigue, and pain [45, 46]. After cytoreductive surgery and HIPEC, one study reported a gradual deterioration of several PROs (global health status, physical functioning, social functioning, emotional functioning, fatigue, and pain) whereas a second study reported that several PROs deteriorated shortly after surgery (physical well-being, functional well-being, fatigue, pain) but recovered quickly and remained stable during follow-up [45, 47]. No direct comparisons of PROs during treatment with PIPAC and during treatment with, e.g., systemic chemotherapy were found. Future studies should focus on this comparison to put the reported changes in PROs during treatment with PIPAC-OX into perspective. 

Together with a French multicenter retrospective cohort study and the safety and feasibility report of the CRC-PIPAC trial [15, 48], the present study suggests that abdominal pain is the most relevant worsening PRO after ePIPAC-OX. Abdominal pain after (e)PIPAC-OX is probably caused by a combination of local pain at trocar sites and diffuse abdominal pain due to chemotherapy-induced chemical peritonitis. This may be drug dependent, as previous reports showed that PIPAC-OX results in a greater inflammatory response and postoperative morphine demand than cisplatin/doxorubicin-based PIPAC [49, 50]. The other worsening gastrointestinal symptoms (i.e., appetite loss, diarrhea, flatulence) may be a result of local chemotherapy-induced changes in gastrointestinal motility. The combination of these worsening symptoms, the effects of general anesthesia, and the (toxicity of) the relevant systemic oxaliplatin uptake after (e)PIPAC-OX [51, 52] could have led to the observed worsening of more general PROs such as fatigue or physical functioning. The role of electrostatic precipitation and the concomitant intravenous administration of 5-fluorouracil/leucovorin in observed PROs and side effects are currently unknown and may be subject of future research.

While the present study assessed ePIPAC-OX monotherapy, several PIPAC centers regularly offer (e)PIPAC-OX in combination with palliative systemic therapy with the aim to maximize intraperitoneal disease control [13]. Three 6-weekly cycles of first-line systemic chemotherapy and bevacizumab followed by ePIPAC-OX (i.e., bidirectional treatment) are currently investigated in 20 patients with isolated unresectable CPM in an ongoing, multicenter, single-arm phase 2 trial (CRC-PIPAC-II,Netherlands Trial Register: NL8303) [53]. In this trial, PROs (calculated from EQ-5D-5L, EORTC QLQ-C30, and EORTC QLQ-CR29) are explored after the first 6 weeks of first-line palliative systemic therapy (before the first ePIPAC-OX) and 1 and 4 weeks after each ePIPAC-OX procedure. Although the population of CRC-PIPAC-II slightly differs from the population of the present study (which also included patients in later lines of palliative treatment), CRC-PIPAC-II may increase insight in the difference in PROs between bidirectional treatment and ePIPAC-OX monotherapy.

Three other single-arm trials are currently assessing PROs during or after (e)PIPAC-OX for unresectable CPM. A Singaporean single-center phase 1 trial assesses EORTC QLQ-C30 at 6 and 12 weeks during 6-weekly PIPAC-OX monotherapy in five dose levels (45 mg/m^2^ to 150 mg/m^2^) in patients with unresectable peritoneal metastases of various origins (including colorectal) who completed, refused, or were unable to tolerate first-line systemic therapy (Clinicaltrials.gov: NCT03172416) [54]. A British single-center phase 2 trial analyzes EORTC QLQ-C30 just before every procedure during 6-to-8-week PIPAC-OX, with or without concomitant systemic chemotherapy therapy, in 30 patients with unresectable CPM in various lines of palliative treatment (Clinicaltrials.gov: NCT03868228). A Danish single-center phase 2 trial assesses EORTC QLQ-C30 at 4 months during (or after) (e)PIPAC with various drugs (including oxaliplatin), with or without concomitant palliative systemic therapy, in patients with unresectable peritoneal metastases of various origins (including cpm) in various lines of palliative treatment (PIPAC-OPC2,Clinicaltrials.gov: NCT03287375) [55]. The international PIPAC registry (Clinicaltrials.gov: NCT03210298), which also analyzes EORTC QLQ-C30, may provide further insight in real-world PROs outside of clinical trials.

The results of the CRC-PIPAC trial, ongoing trials, and the international PIPAC registry may be used to design future randomized trials to determine the role of (e)PIPAC-OX in the treatment of patients with isolated (initially) unresectable CPM. Importantly, international consensus must be reached on the most appropriate interventions (e.g., PIPAC-OX monotherapy, bidirectional treatment), settings (e.g., neoadjuvant, first-line palliative, refractory), frameworks (e.g., superiority, equivalence, non-inferiority), and endpoints (e.g., survival, PROs, combination of both) of such trials. If PROs will be used as a primary endpoint, the results of the present study and ongoing trials may be used to choose the most appropriate PROs and may serve as a basis for sample size calculations.

The small sample size was the main limitation of this explorative study. A larger sample size may have detected statistically significant fluctuations in PROs that could have been clinically relevant. Furthermore, while questionnaire response rates were high, trial treatment was stopped before the second procedure in four of twenty patients, and before the third procedure in an additional three patients. Although inevitable in trials including patients with a very poor prognosis, this drop-out reduced the statistical power of comparisons of baseline scores with scores after the second and third procedures. Nevertheless, despite the small sample size, linear mixed modeling analyses allowed for the detection of both statistically significant and clinically relevant findings.

## Conclusions

Patients with isolated unresectable CPM receiving repetitive ePIPAC-OX monotherapy (i.e., without palliative systemic therapy in between subsequent procedures) had clinically relevant but reversible worsening of several PROs during trial treatment, mainly after the first procedure. All worsening PROs eventually returned to baseline 4 weeks after the third procedure. Despite these changes in several PROs, it should also be noted that all other analyzed PROs did not change during trial treatment. The results of the present study may be used to inform patients about the burden of (e)PIPAC-OX, should be taken into account by physicians when proposing (e)PIPAC-OX in the palliative setting, and could help designing future PRO-focused randomized trials to determine the role of (e)PIPAC-OX in the palliative treatment of CPM.

## Supplementary Information

Below is the link to the electronic supplementary material.

Supplementary file1 (DOCX 30 kb)

Supplementary file2 (DOCX 33 kb)

Supplementary file3 (PDF 884 kb)

Supplementary file4 (PDF 649 kb)
