# Supplementary material for: Patient-reported outcomes during repetitive oxaliplatin-based pressurized intraperitoneal aerosol chemotherapy for isolated unresectable colorectal peritoneal metastases in a multicenter, single-arm, phase 2 trial (CRC-PIPAC)
Source: Surg Endosc. 2021 Nov 10;36(6):4486–98. doi: 10.1007/s00464-021-08802-6 (PMC9085665; doi:10.1007/s00464-021-08802-6)
Supplement: Supplementary file 1 — Supplementary file1 (DOCX 30 kb) [file 464_2021_8802_MOESM1_ESM.docx]

**Appendix A.** Linear mixed modeling analyses of 12 PROs with a statistically significant difference in scores between baseline and at least one subsequent time point.

| **Function scales** | | | | | | |  |  |  |
| --- | --- | --- | --- | --- | --- | --- | --- | --- | --- |
| **PRO** | **Mean difference^a^** | | | | **95% CI** | | **p-value** | **Cohen’s d^b^** | **Degree of deterioration** |
| **Index value (EQ-5D-5L)** | | | | | | |  |  |  |
| Baseline vs. one week after first procedure | -0.10 | | | | -0.16 – -0.05 | | **<0.001** | **0.76** | MID |
| Baseline vs. four weeks after first procedure | -0.02 | | | | -0.09 – 0.02 | | 0.24 | - |  |
| Baseline vs. one week after second procedure | -0.03 | | | | -0.10 – 0.02 | | 0.18 | - |  |
| Baseline vs. four weeks after second procedure | -0.01 | | | | -0.09 – 0.03 | | 0.35 | - |  |
| Baseline vs. one week after third procedure | -0.04 | | | | -0.12 – 0.01 | | 0.12 | - |  |
| Baseline vs. four weeks after third procedure | +0.01 | | | | -0.06 – 0.06 | | 0.99 | - |  |
| **Physical functioning (EORTC QLQ-C30)** | | | | | | |  |  |  |
| Baseline vs. one week after first procedure | -20 | | | | -27 – -12 | | **<0.001** | **1.03** | Major |
| Baseline vs. four weeks after first procedure | -6 | | | | -14 – 2 | | 0.12 | - |  |
| Baseline vs. one week after second procedure | -11 | | | | -19 – -3 | | 0.0085 | **-** |  |
| Baseline vs. four weeks after second procedure | -6 | | | | -13 – 3 | | 0.19 | - |  |
| Baseline vs. one week after third procedure | -9 | | | | -18 – -1 | | 0.03 | - |  |
| Baseline vs. four weeks after third procedure | -1 | | | | -11 – 6 | | 0.58 | - |  |
| **Role functioning (EORTC QLQ-C30)** | | | | | | |  |  |  |
| Baseline vs. one week after first procedure | -27 | | | | -39 – -15 | | **<0.001** | **0.87** | Major |
| Baseline vs. four weeks after first procedure | +0 | | | | -13 – 12 | | 0.97 | - |  |
| Baseline vs. one week after second procedure | -16 | | | | -30 – -4 | | 0.01 | - |  |
| Baseline vs. four weeks after second procedure | -5 | | | | -19 – 7 | | 0.35 | - |  |
| Baseline vs. one week after third procedure | -13 | | | | -29 – 0 | | 0.05 | - |  |
| Baseline vs. four weeks after third procedure | +7 | | | | -8 – 21 | | 0.37 | - |  |
| **Social functioning (EORTC QLQ-C30)** | | | | | | |  |  |  |
| Baseline vs. one week after first procedure | -18 | | | | -28 – -8 | | **<0.001** | **0.71** | Moderate |
| Baseline vs. four weeks after first procedure | -1 | | | | -12 – 9 | | 0.75 | - |  |
| Baseline vs. one week after second procedure | -1 | | | | -12 – 9 | | 0.76 | - |  |
| Baseline vs. four weeks after second procedure | +1 | | | | -10 – 11 | | 0.94 | - |  |
| Baseline vs. one week after third procedure | -4 | | | | -16 – 7 | | 0.46 | - |  |
| Baseline vs. four weeks after third procedure | +5 | | | | -8 – 16 | | 0.51 | - |  |
| **C30 summary score (EORTC QLQ-C30)** | | | | | | |  |  |  |
| Baseline vs. one week after first procedure | -16 | | | | -20 – -9 | | **<0.001** | **1.07** | Moderate |
| Baseline vs. four weeks after first procedure | -2 | | | | -9 – 2 | | 0.24 | - |  |
| Baseline vs. one week after second procedure | -7 | | | | -13 – -1 | | 0.02 | - |  |
| Baseline vs. four weeks after second procedure | -1 | | | | -7 – 5 | | 0.78 | - |  |
| Baseline vs. one week after third procedure | -6 | | | | -14 – -1 | | 0.03 | - |  |
| Baseline vs. four weeks after third procedure | +2 | | | | -6 – 7 | | 0.89 | - |  |
| **Symptom scales** | | | | | | |  |  |  |
| **PRO** | **Mean difference^a^** | | | | **95% CI** | | **p-value** | **Cohen’s d^b^** | **Degree of deterioration** |
| **Fatigue (EORTC QLQ-C30)** | | | | | | |  |  |  |
| Baseline vs. one week after first procedure | +23 | | | | 14 – 33 | | **<0.001** | **0.98** | Major |
| Baseline vs. four weeks after first procedure | +7 | | | | -2 – 18 | | 0.13 | - |  |
| Baseline vs. one week after second procedure | +20 | | | | 9 – 30 | | **<0.001** | **0.83** | Major |
| Baseline vs. four weeks after second procedure | +5 | | | | -5 – 16 | | 0.32 | - |  |
| Baseline vs. one week after third procedure | +13 | | | | 3 – 26 | | 0.02 | - |  |
| Baseline vs. four weeks after third procedure | +3 | | | | -7 – 16 | | 0.41 | - |  |
| **Pain (EORTC QLQ-C30)** | | | | | | |  |  |  |
| Baseline vs. one week after first procedure | +29 | | | | 19 – 40 | | **<0.001** | **1.49** | Major |
| Baseline vs. four weeks after first procedure | +8 | | | | -4 – 18 | | 0.20 | - |  |
| Baseline vs. one week after second procedure | +21 | | | | 10 – 33 | | **<0.001** | **0.95** | Major |
| Baseline vs. four weeks after second procedure | +8 | | | | -2 – 21 | | 0.11 | - |  |
| Baseline vs. one week after third procedure | +22 | | | | 11 – 36 | | **<0.001** | **0.95** | Major |
| Baseline vs. four weeks after third procedure | +4 | | | | -7 – 18 | | 0.36 | - |  |
| **Appetite loss (EORTC QLQ-C30)** | | | | | | |  |  |  |
| Baseline vs. one week after first procedure | +15 | | | | 4 – 26 | | **0.007** | 0.43 | Moderate |
| Baseline vs. four weeks after first procedure | -1 | | | | -11 – 12 | | 0.92 | - |  |
| Baseline vs. one week after second procedure | +12 | | | | 1 – 24 | | 0.04 | - |  |
| **Symptom scales (continued…)** | | | | | | | | |  |
| **PRO** | |  |  | **Mean difference^a^** | | **95% CI** | **p-value** | **Cohen’s d^b^** | **Degree of deterioration** |
| Baseline vs. four weeks after second procedure | +2 | | | | -10 – 14 | | 0.76 | - |  |
| Baseline vs. one week after third procedure | +14 | | | | 0 – 25 | | 0.05 | - |  |
| Baseline vs. four weeks after third procedure | +0 | | | | -14 – 12 | | 0.86 | - |  |
| **Diarrhea (EORTC QLQ-C30)** | | | | | | |  |  |  |
| Baseline vs. one week after first procedure | +15 | | | | 5 – 25 | | **0.002** | **0.65** | Minor |
| Baseline vs. four weeks after first procedure | +7 | | | | -2 – 18 | | 0.12 | - |  |
| Baseline vs. one week after second procedure | -6 | | | | -15 – 5 | | 0.32 | - |  |
| Baseline vs. four weeks after second procedure | -2 | | | | -11 – 9 | | 0.85 | - |  |
| Baseline vs. one week after third procedure | +5 | | | | -7 – 15 | | 0.47 | - |  |
| Baseline vs. four weeks after third procedure | +2 | | | | -10 – 13 | | 0.81 | - |  |
| **Urinary frequency (EORTC QLQ-CR29)** | | | | | | |  |  |  |
| Baseline vs. one week after first procedure | +13 | | | | 4 – 22 | | **0.004** | **0.74** | Moderate |
| Baseline vs. four weeks after first procedure | +12 | | | | 2 – 21 | | 0.02 | - |  |
| Baseline vs. one week after second procedure | +14 | | | | 3 – 22 | | 0.01 | - |  |
| Baseline vs. four weeks after second procedure | +9 | | | | -3 – 17 | | 0.16 | - |  |
| Baseline vs. one week after third procedure | +14 | | | | 3 – 24 | | 0.01 | - |  |
| Baseline vs. four weeks after third procedure | +7 | | | | -4 – 17 | | 0.22 | - |  |
| **Abdominal pain (EORTC QLQ-CR29)** | | | | | | |  |  |  |
| Baseline vs. one week after first procedure | +32 | | | | 20 – 43 | | **<0.001** | **1.58** | Major |
| Baseline vs. four weeks after first procedure | +19 | | | | 7 – 31 | | **0.003** | **0.89** | Moderate |
| Baseline vs. one week after second procedure | +20 | | | | 7 – 33 | | **0.002** | **0.86** | Moderate |
| Baseline vs. four weeks after second procedure | +15 | | | | 3 – 29 | | 0.02 | - |  |
| Baseline vs. one week after third procedure | +22 | | | | 9 – 36 | | **0.002** | **1.03** | Major |
| Baseline vs. four weeks after third procedure | +2 | | | | -11 – 17 | | 0.67 | - |  |
| **Flatulence (EORTC QLQ-CR29)** | | | | | | |  |  |  |
| Baseline vs. one week after first procedure | +13 | | | | 6 – 21 | | **0.001** | **0.56** | Moderate |
| Baseline vs. four weeks after first procedure | +4 | | | | -4 – 12 | | 0.29 | - |  |
| Baseline vs. one week after second procedure | -2 | | | | -9 – 7 | | 0.75 | - |  |
| Baseline vs. four weeks after second procedure | -9 | | | | -16 – 1 | | 0.07 | - |  |
| Baseline vs. one week after third procedure | -3 | | | | -13 – 5 | | 0.35 | - |  |
| Baseline vs. four weeks after third procedure | -3 | | | | -13 – 5 | | 0.35 | - |  |
| *CI* confidence interval; *MID* minimally important difference; *PRO* patient-reported outcome; ^a^calculated as the mean score of a time point minus mean baseline score; ^b^calculated as mean difference divided by pooled standard deviation. | | | | | | | | |  |
