# Supplementary material for: Patient-reported outcomes during repetitive oxaliplatin-based pressurized intraperitoneal aerosol chemotherapy for isolated unresectable colorectal peritoneal metastases in a multicenter, single-arm, phase 2 trial (CRC-PIPAC)
Source: Surg Endosc. 2021 Nov 10;36(6):4486–98. doi: 10.1007/s00464-021-08802-6 (PMC9085665; doi:10.1007/s00464-021-08802-6)
Supplement: Supplementary file 2 — Supplementary file2 (DOCX 33 kb) [file 464_2021_8802_MOESM2_ESM.docx]

**Appendix B.** Linear mixed modeling analyses of 29 PROs without a statistically significant difference in scores between baseline and subsequent time points.

| **Function scales** | | | |
| --- | --- | --- | --- |
| **PRO** | **Mean difference^a^** | **95% CI** | **p-value** |
| **Visual analogue scale (EQ-5D-5L)** | | | |
| Baseline vs. one week after first procedure | -8 | -22 – 5 | 0.22 |
| Baseline vs. four weeks after first procedure | +2 | -12 – 16 | 0.79 |
| Baseline vs. one week after second procedure | +2 | -13 – 17 | 0.80 |
| Baseline vs. four weeks after second procedure | -3 | -17 – 12 | 0.72 |
| Baseline vs. one week after third procedure | +4 | -14 – 19 | 0.76 |
| Baseline vs. four weeks after third procedure | +1 | -17 – 15 | 0.94 |
| **Global health status (EORTC QLQ-C30)** | | | |
| Baseline vs. one week after first procedure | -8 | -17 – -1 | 0.03 |
| Baseline vs. four weeks after first procedure | +2 | -5 – 10 | 0.54 |
| Baseline vs. one week after second procedure | -5 | -12 – 4 | 0.36 |
| Baseline vs. four weeks after second procedure | 0 | -7 – 10 | 0.73 |
| Baseline vs. one week after third procedure | -2 | -10 – 8 | 0.84 |
| Baseline vs. four weeks after third procedure | +2 | -6 – 12 | 0.48 |
| **Emotional functioning (EORTC QLQ-C30)** | | | |
| Baseline vs. one week after first procedure | -1 | -7 – 5 | 0.79 |
| Baseline vs. four weeks after first procedure | +2 | -6 – 7 | 0.82 |
| Baseline vs. one week after second procedure | -5 | -11 – 2 | 0.19 |
| Baseline vs. four weeks after second procedure | -1 | -6 – 7 | 0.90 |
| Baseline vs. one week after third procedure | -1 | -6 – 8 | 0.76 |
| Baseline vs. four weeks after third procedure | -3 | -9 – 6 | 0.65 |
| **Cognitive functioning (EORTC QLQ-C30)** | | | |
| Baseline vs. one week after first procedure | -6 | -13 – 1 | 0.11 |
| Baseline vs. four weeks after first procedure | +3 | -6 – 9 | 0.64 |
| Baseline vs. one week after second procedure | -1 | -9 – 7 | 0.81 |
| Baseline vs. four weeks after second procedure | +8 | 0 – 15 | 0.06 |
| Baseline vs. one week after third procedure | +2 | -6 – 11 | 0.49 |
| Baseline vs. four weeks after third procedure | +2 | -6 – 11 | 0.49 |
| **Body image (EORTC QLQ-CR29)** | | | |
| Baseline vs. one week after first procedure | -4 | -10 – 3 | 0.31 |
| Baseline vs. four weeks after first procedure | +3 | -5 – 8 | 0.70 |
| Baseline vs. one week after second procedure | -3 | -10 – 4 | 0.35 |
| Baseline vs. four weeks after second procedure | -3 | -10 – 4 | 0.45 |
| Baseline vs. one week after third procedure | -5 | -11 – 5 | 0.42 |
| Baseline vs. four weeks after third procedure | -6 | -13 – 3 | 0.20 |
| **Weight (EORTC QLQ-CR29)** | | | |
| Baseline vs. one week after first procedure | -3 | -14 – 7 | 0.51 |
| Baseline vs. four weeks after first procedure | +4 | -10 – 11 | 0.89 |
| Baseline vs. one week after second procedure | +3 | -9 – 13 | 0.71 |
| Baseline vs. four weeks after second procedure | -5 | -17 – 5 | 0.25 |
| Baseline vs. one week after third procedure | -5 | -18 – 6 | 0.30 |
| Baseline vs. four weeks after third procedure | -2 | -15 – 8 | 0.56 |
| **Anxiety (EORTC QLQ-CR29)** | | | |
| Baseline vs. one week after first procedure | +9 | -1 – 18 | 0.09 |
| Baseline vs. four weeks after first procedure | +7 | -4 – 16 | 0.22 |
| Baseline vs. one week after second procedure | +3 | -5 – 16 | 0.32 |
| Baseline vs. four weeks after second procedure | +7 | -1 – 20 | 0.07 |
| Baseline vs. one week after third procedure | +3 | -8 – 14 | 0.61 |
| Baseline vs. four weeks after third procedure | +8 | -3 – 20 | 0.14 |
| **Sexual interest, males (EORTC QLQ-CR29)** | | | |
| Baseline vs. one week after first procedure | -9 | -16 – 0 | 0.05 |
| Baseline vs. four weeks after first procedure | -6 | -14 – 3 | 0.18 |
| Baseline vs. one week after second procedure | -9 | -11 – 7 | 0.59 |
| Baseline vs. four weeks after second procedure | -12 | -15 – 3 | 0.18 |
| Baseline vs. one week after third procedure | -10 | -16 – 3 | 0.19 |
| Baseline vs. four weeks after third procedure | -6 | -11 – 7 | 0.66 |
| **Sexual interest, females (EORTC QLQ-CR29)** | | | |
| Baseline vs. one week after first procedure | -4 | -11 – 3 | 0.25 |
| Baseline vs. four weeks after first procedure | +2 | -7 – 8 | 0.90 |
| Baseline vs. one week after second procedure | -3 | -12 – 3 | 0.26 |
| Baseline vs. four weeks after second procedure | +2 | -7 – 8 | 0.90 |
| Baseline vs. one week after third procedure | -8 | -19 – -1 | 0.04 |
| Baseline vs. four weeks after third procedure | 0 | -10 – 8 | 0.76 |
| **Symptom scales** | | | |
| **PRO** | **Mean difference^a^** | **95% CI** | **p-value** |
| **Nausea/vomiting (EORTC QLQ-C30)** | | | |
| Baseline vs. one week after first procedure | +6 | -2 – 15 | 0.11 |
| Baseline vs. four weeks after first procedure | -5 | -10 – 7 | 0.80 |
| Baseline vs. one week after second procedure | -2 | -8 – 10 | 0.80 |
| Baseline vs. four weeks after second procedure | -6 | -11 – 7 | 0.64 |
| Baseline vs. one week after third procedure | +1 | -5 – 14 | 0.33 |
| Baseline vs. four weeks after third procedure | -7 | -13 – 6 | 0.47 |
| **Dyspnea (EORTC QLQ-C30)** | | | |
| Baseline vs. one week after first procedure | +1 | -7 – 10 | 0.71 |
| Baseline vs. four weeks after first procedure | -3 | -11 – 7 | 0.66 |
| Baseline vs. one week after second procedure | +3 | -6 – 12 | 0.52 |
| Baseline vs. four weeks after second procedure | -6 | -15 – 4 | 0.26 |
| Baseline vs. one week after third procedure | -4 | -9 – 12 | 0.73 |
| Baseline vs. four weeks after third procedure | -6 | -11 – 9 | 0.85 |
| **Insomnia (EORTC QLQ-C30)** | | | |
| Baseline vs. one week after first procedure | +6 | -7 – 20 | 0.32 |
| Baseline vs. four weeks after first procedure | +7 | -4 – 23 | 0.18 |
| Baseline vs. one week after second procedure | +14 | 1 – 29 | 0.04 |
| Baseline vs. four weeks after second procedure | +2 | -12 – 17 | 0.72 |
| Baseline vs. one week after third procedure | 0 | -12 – 19 | 0.63 |
| Baseline vs. four weeks after third procedure | -3 | -15 – 17 | 0.89 |
| **Constipation (EORTC QLQ-C30)** | | | |
| Baseline vs. one week after first procedure | +14 | 2 – 25 | 0.03 |
| Baseline vs. four weeks after first procedure | +5 | -8 – 16 | 0.49 |
| Baseline vs. one week after second procedure | +4 | -9 – 16 | 0.57 |
| Baseline vs. four weeks after second procedure | -2 | -15 – 10 | 0.68 |
| Baseline vs. one week after third procedure | +3 | -12 – 15 | 0.87 |
| Baseline vs. four weeks after third procedure | -5 | -21 – 6 | 0.30 |
| **Financial difficulties (EORTC QLQ-C30)** | | | |
| Baseline vs. one week after first procedure | +7 | 1 – 12 | 0.02 |
| Baseline vs. four weeks after first procedure | -1 | -5 – 6 | 0.93 |
| Baseline vs. one week after second procedure | -1 | -3 – 9 | 0.28 |
| Baseline vs. four weeks after second procedure | -3 | -5 – 7 | 0.70 |
| Baseline vs. one week after third procedure | +3 | -3 -10 | 0.27 |
| Baseline vs. four weeks after third procedure | +3 | -3 – 10 | 0.27 |
| **Urinary incontinence (EORTC QLQ-CR29)** | | | |
| Baseline vs. one week after first procedure | -2 | -6 – 3 | 0.46 |
| Baseline vs. four weeks after first procedure | +2 | -3 – 6 | 0.43 |
| Baseline vs. one week after second procedure | +2 | -2 – 7 | 0.34 |
| Baseline vs. four weeks after second procedure | +2 | -2 – 7 | 0.32 |
| Baseline vs. one week after third procedure | +1 | -4 – 6 | 0.70 |
| Baseline vs. four weeks after third procedure | -2 | -7 – 4 | 0.51 |
| **Dysuria (EORTC QLQ-CR29)** | | | |
| Baseline vs. one week after first procedure | +4 | -4 – 11 | 0.38 |
| Baseline vs. four weeks after first procedure | +4 | -4 – 11 | 0.36 |
| Baseline vs. one week after second procedure | +3 | -6 – 10 | 0.62 |
| Baseline vs. four weeks after second procedure | +7 | -1 – 14 | 0.13 |
| Baseline vs. one week after third procedure | +3 | -9 – 9 | 0.95 |
| Baseline vs. four weeks after third procedure | +3 | -9 – 9 | 0.95 |
| **Buttock pain (EORTC QLQ-CR29)** | | | |
| Baseline vs. one week after first procedure | -2 | -9 – 6 | 0.65 |
| Baseline vs. four weeks after first procedure | +4 | -2 – 13 | 0.16 |
| Baseline vs. one week after second procedure | -1 | -8 – 8 | 0.96 |
| Baseline vs. four weeks after second procedure | -1 | -8 – 8 | 0.96 |
| Baseline vs. one week after third procedure | +1 | -7 – 10 | 0.67 |
| Baseline vs. four weeks after third procedure | -4 | -12 – 5 | 0.40 |
| **Bloating (EORTC QLQ-CR29)** | | | |
| Baseline vs. one week after first procedure | +7 | -6 – 20 | 0.31 |
| Baseline vs. four weeks after first procedure | -5 | -18 – 8 | 0.46 |
| Baseline vs. one week after second procedure | +5 | -9 – 19 | 0.50 |
| Baseline vs. four weeks after second procedure | +1 | -13 – 15 | 0.93 |
| Baseline vs. one week after third procedure | 0 | -14 – 17 | 0.82 |
| Baseline vs. four weeks after third procedure | -6 | -19 – 12 | 0.63 |
| **Blood/mucus in stool (EORTC QLQ-CR29)** | | | |
| Baseline vs. one week after first procedure | +3 | 0 – 7 | 0.05 |
| Baseline vs. four weeks after first procedure | +3 | -1 – 6 | 0.12 |
| Baseline vs. one week after second procedure | +2 | -2 – 5 | 0.27 |
| **Symptom scales (continued…)** | | | |
| **PRO** | **Mean difference^a^** | **95% CI** | **p-value** |
| Baseline vs. four weeks after second procedure | +3 | 0 – 6 | 0.09 |
| Baseline vs. one week after third procedure | +1 | -3 – 5 | 0.52 |
| Baseline vs. four weeks after third procedure | 0 | -4 – 4 | 0.95 |
| **Dry mouth (EORTC QLQ-CR29)** | | | |
| Baseline vs. one week after first procedure | +9 | -2 – 18 | 0.10 |
| Baseline vs. four weeks after first procedure | +6 | -2 – 19 | 0.10 |
| Baseline vs. one week after second procedure | +7 | -3 – 18 | 0.16 |
| Baseline vs. four weeks after second procedure | +1 | -9 – 12 | 0.81 |
| Baseline vs. one week after third procedure | +4 | -4 – 20 | 0.18 |
| Baseline vs. four weeks after third procedure | -1 | -9 – 14 | 0.68 |
| **Hair loss (EORTC QLQ-CR29)** | | | |
| Baseline vs. one week after first procedure | +2 | -5 – 9 | 0.64 |
| Baseline vs. four weeks after first procedure | +2 | -5 – 9 | 0.62 |
| Baseline vs. one week after second procedure | +1 | -8 – 8 | 0.99 |
| Baseline vs. four weeks after second procedure | +5 | -3 – 12 | 0.28 |
| Baseline vs. one week after third procedure | +5 | -3 – 13 | 0.25 |
| Baseline vs. four weeks after third procedure | +8 | -1 – 16 | 0.07 |
| **Taste (EORTC QLQ-CR29)** | | | |
| Baseline vs. one week after first procedure | +4 | -6 – 12 | 0.46 |
| Baseline vs. four weeks after first procedure | -2 | -9 – 9 | 0.95 |
| Baseline vs. one week after second procedure | +9 | -1 – 19 | 0.07 |
| Baseline vs. four weeks after second procedure | +5 | -5 – 14 | 0.34 |
| Baseline vs. one week after third procedure | +1 | -8 – 13 | 0.61 |
| Baseline vs. four weeks after third procedure | -4 | -13 – 8 | 0.60 |
| **Fecal incontinence (EORTC QLQ-CR29)** | | | |
| Baseline vs. one week after first procedure | 0 | -5 – 5 | >0.99 |
| Baseline vs. four weeks after first procedure | -1 | -7 – 4 | 0.67 |
| Baseline vs. one week after second procedure | -3 | -9 – 3 | 0.30 |
| Baseline vs. four weeks after second procedure | -3 | -9 – 3 | 0.30 |
| Baseline vs. one week after third procedure | -2 | -8 – 4 | 0.52 |
| Baseline vs. four weeks after third procedure | -5 | -11 – 1.5 | 0.13 |
| **Sore skin (EORTC QLQ-CR29)** | | | |
| Baseline vs. one week after first procedure | +3 | -3 – 10 | 0.32 |
| Baseline vs. four weeks after first procedure | +4 | -3 – 11 | 0.22 |
| Baseline vs. one week after second procedure | -5 | -10 – 4 | 0.44 |
| Baseline vs. four weeks after second procedure | -3 | -8 – 6 | 0.84 |
| Baseline vs. one week after third procedure | -1 | -8 – 8 | 0.99 |
| Baseline vs. four weeks after third procedure | -1 | -8 – 8 | 0.99 |
| **Stool frequency (EORTC QLQ-CR29)** | | | |
| Baseline vs. one week after first procedure | +7 | 1 – 14 | 0.03 |
| Baseline vs. four weeks after first procedure | -3 | -7 – 6 | 0.85 |
| Baseline vs. one week after second procedure | -1 | -6 – 8 | 0.82 |
| Baseline vs. four weeks after second procedure | -2 | -7 – 7 | 0.96 |
| Baseline vs. one week after third procedure | -1 | -7 – 8 | 0.87 |
| Baseline vs. four weeks after third procedure | -1 | -7 – 8 | 0.87 |
| **Embarrassment (EORTC QLQ-CR29)** | | | |
| Baseline vs. one week after first procedure | 0 | -8 – 8 | >0.99 |
| Baseline vs. four weeks after first procedure | -3 | -11 – 6 | 0.51 |
| Baseline vs. one week after second procedure | 0 | -9 – 9 | 0.93 |
| Baseline vs. four weeks after second procedure | -6 | -15 – 3 | 0.20 |
| Baseline vs. one week after third procedure | -7 | -16 – 4 | 0.23 |
| Baseline vs. four weeks after third procedure | -7 | -16 – 4 | 0.23 |
| **Stoma care problems (EORTC QLQ-CR29)** | | | |
| Baseline vs. one week after first procedure | 0 | -13 – 13 | >0.99 |
| Baseline vs. four weeks after first procedure | -8 | -22 – 5 | 0.21 |
| Baseline vs. one week after second procedure | -9 | -14 – 21 | 0.67 |
| Baseline vs. four weeks after second procedure | +25 | 3 – 37 | 0.02 |
| Baseline vs. one week after third procedure | +9 | -14 – 21 | 0.67 |
| Baseline vs. four weeks after third procedure | +9 | -14 – 21 | 0.67 |
| **Impotence, males (EORTC QLQ-CR29)** | | | |
| Baseline vs. one week after first procedure | +12 | 1 – 24 | 0.04 |
| Baseline vs. four weeks after first procedure | +9 | -2 – 21 | 0.12 |
| Baseline vs. one week after second procedure | +5 | -9 – 16 | 0.59 |
| Baseline vs. four weeks after second procedure | +5 | -9 – 16 | 0.59 |
| Baseline vs. one week after third procedure | +10 | -5 – 20 | 0.23 |
| Baseline vs. four weeks after third procedure | +1 | -13 – 12 | 0.92 |
| **Dyspareunia, females (EORTC QLQ-CR29)** | | | |
| **Symptom scales (continued…)** | | | |
| **PRO** | **Mean difference^a^** | **95% CI** | **p-value** |
| Baseline vs. one week after first procedure | -4 | -8 – 6 | 0.74 |
| Baseline vs. four weeks after first procedure | -4 | -9 – 6 | 0.76 |
| Baseline vs. one week after second procedure | -4 | -9 – 6 | 0.76 |
| Baseline vs. four weeks after second procedure | +6 | -2 – 12 | 0.17 |
| Baseline vs. one week after third procedure | +7 | -1 – 19 | 0.07 |
| Baseline vs. four weeks after third procedure | +13 | 0 – 17 | 0.05 |
| *CI* confidence interval; *PRO* patient-reported outcome; ^a^calculated as mean score of a time point minus mean baseline score. | | | |
