# Supplementary material for: Patient-reported outcomes during repetitive oxaliplatin-based pressurized intraperitoneal aerosol chemotherapy for isolated unresectable colorectal peritoneal metastases in a multicenter, single-arm, phase 2 trial (CRC-PIPAC)
Source: Surg Endosc. 2021 Nov 10;36(6):4486–98. doi: 10.1007/s00464-021-08802-6 (PMC9085665; doi:10.1007/s00464-021-08802-6)
Supplement: Supplementary file 3 — Supplementary file3 (PDF 884 kb) [file 464_2021_8802_MOESM3_ESM.pdf]

Protocol of the CRC-PIPAC study

ISRCTN identifier ISRCTN89947480; ClinicalTrials.gov identifier NCT03246321;
EudraCT identifier 2017-000927-29; NTR identifier NTR6603;

PROTOCOL TITLE 'Repetitive electrostatic pressurised intraperitoneal aerosol chemotherapy with oxaliplatin as a palliative monotherapy for isolated unresectable colorectal peritoneal metastases: protocol of a multicentre, open-label, single-arm, phase II study (CRC-PIPAC)'

**Protocol ID**	NL60405.100.17
**Short title**	CRC-PIPAC
**EudraCT number**	2017-000927-29
**Version**	6
**Date**	11-01-2019
**Coordinating investigator**	Koen P. Rovers, MD.<br>Department of Surgery, Catharina Hospital<br>Michelangelolaan 2, 5623 EJ, Eindhoven, Netherlands<br>koen.rovers@catharinaziekenhuis.nl<br>+31 40 239 6351
**Project leader**	Ignace H.J.T. de Hingh, MD, PhD<br>Department of Surgery, Catharina Hospital<br>Michelangelolaan 2, 5623 EJ, Eindhoven, Netherlands<br>ignace.d.hingh@catharinaziekenhuis.nl<br>+31 40 239 7150
**Principal investigators**	Djamila Boerma, MD, PhD<br>Department of Surgery, St. Antonius Hospital<br>Koekoekslaan 1, 3435 CM, Nieuwegein, Netherlands<br>d.boerma@antoniusziekenhuis.nl<br>+31 88 320 1900<br><br>Ignace H.J.T. de Hingh, MD, PhD<br>Department of Surgery, Catharina Hospital<br>Michelangelolaan 2, 5623 EJ, Eindhoven, Netherlands

	ignace.d.hingh@catharinaziekenhuis.nl<br>+31 40 239 7150
**Sponsor**	Catharina Hospital<br>Michelangelolaan 2, 5623 EJ, Eindhoven, Netherlands<br>+31 40 239 9111
**Subsidising parties**	Catharina Research Fund<br>Michelangelolaan 2, 5623 EJ, Eindhoven, Netherlands<br>+31 40 239 8480<br><br>St. Antonius Research Fund<br>Koekoekslaan 1, 3435 CM, Nieuwegein, Netherlands<br>+31 88 320 8815
**Independent expert**	Erik J. Schoon, MD, PhD<br>Department of Gastroenterology, Catharina Hospital<br>Michelangelolaan 2, 5623 EJ, Eindhoven, Netherlands<br>erik.schoon@catharinaziekenhuis.nl<br>+31 40 239 9750
**Central laboratory**	Remond J.A. Fijneman, PhD<br>Department of Pathology, Netherlands Cancer Institute<br>Plesmanlaan 121, 1066 CX, Amsterdam, Netherlands<br>r.fijneman@nki.nl<br>+31 20 512 9111
**Pharmacy**	Maarten J. Deenen, PhD<br>Department of Clinical Pharmacy, Catharina Hospital<br>Michelangelolaan 2, 5623 EJ, Eindhoven, Netherlands<br>maarten.deenen@catharinaziekenhuis.nl

PROTOCOL SIGNATURE SHEET

**Name**	**Signature**	**Date**
**Project leader**<br>Ignace H.J.T. de Hingh, MD, PhD<br>Department of Surgery<br>Catharina Hospital, Eindhoven, Netherlands<br>ignace.d.hingh@catharinaziekenhuis.nl<br>+31 40 239 7150		
**Coordinating investigator**<br>Koen P. Rovers, MD<br>Department of Surgery<br>Catharina Hospital, Eindhoven, Netherlands<br>koen.rovers@catharinaziekenhuis.nl<br>+31 40 239 6351		

PROTOCOL COLLABORATORS

Koen P. Rovers[1], Robin J. Lurvink[1], Emma C.E. Wassenaar[2], Thomas J.M. Kootstra[2], Harm J. Scholten[3] Rudaba Tajzai[1,4]; Maarten J. Deenen[4]; Joost Nederend[5]; Max J. Lahaye[6]; Clément J.R. Huysentruyt[7]; Iris van 't Erve[8]; Remond J.A. Fijneman[8]; Alexander Constantinides[9]; Onno Kranenburg[9]; Maartje Los[10]; Anna M.J. Thijs[11]; Geert-Jan M. Creemers[11]; Jacobus W.A. Burger[1]; Marinus J. Wiezer[2]; Djamila Boerma[2]; Simon W. Nienhuijs[1]; Ignace H.J.T. de Hingh[1].

AFFILIATIONS

[1]Surgery, Catharina Hospital, PO Box 1350, 5602 ZA, Eindhoven, Netherlands;

[2]Surgery, St. Antonius Hospital, PO Box 2500, 3430 EM, Nieuwegein, Netherlands;

[3]Anaesthesiology, Catharina Hospital, PO Box 1350, 5602 ZA, Eindhoven, Netherlands;

[4]Clinical Pharmacy, Catharina Hospital, PO Box 1350, 5602 ZA, Eindhoven, Netherlands;

[5]Radiology, Catharina Hospital, PO Box 1350, 5602 ZA, Eindhoven, Netherlands;

[6]Radiology, Netherlands Cancer Institute, PO Box 90203, 1006 BE, Amsterdam, Netherlands;

[7]Pathology, Catharina Hospital, PO Box 1350, 5602 ZA, Eindhoven, Netherlands;

[8]Pathology, Netherlands Cancer Institute, PO Box 90203, 1006 BE, Amsterdam, Netherlands;

[9]Cancer Centre, University Medical Centre Utrecht, PO Box 85500, 3508 GA, Utrecht, Netherlands;

[10]Medical Oncology, St. Antonius Hospital, PO Box 2500, 3430 EM, Nieuwegein, Netherlands;

[11]Medical Oncology, Catharina Hospital, PO Box 1350, 5602 ZA, Eindhoven, Netherlands.

TABLE OF CONTENTS

1. INTRODUCTION AND RATIONALE 10
1.1 PIPAC 11
1.2 PIPAC FOR COLORECTAL PM 12
1.3 RATIONALE FOR THIS STUDY 12
1.4 RATIONALE FOR INTERVENTION 13
2. OBJECTIVES 13
2.1 PRIMARY OBJECTIVE 13
2.2 SECONDARY OBJECTIVES 14
3. STUDY DESIGN 14
3.1 DESIGN 14
3.2 SETTING 14
3.3 DURATION 14
4. STUDY POPULATION 15
4.1 POPULATION (BASE) 15
4.2 ELIGIBILITY CRITERIA 15
4.3 SAMPLE SIZE 16
5. TREATMENT OF SUBJECTS 16
5.1 ePIPAC-OX 16
5.2 OUTPATIENT EVALUATIONS 19
5.3 QUESTIONNAIRES 20
5.4 PHARMACOKINETICS 20
5.5 TRANSLATIONAL RESEARCH 21
6. INVESTIGATIONAL PRODUCT 21
6.1 OXALIPLATIN 21
6.1.1 NAME AND DESCRIPTION 21
6.1.2 SUMMARY OF FINDINGS OF NON-CLINICAL STUDIES 21
6.1.3 SUMMARY OF FINDINGS OF CLINICAL STUDIES 22
6.1.4 SUMMARY OF KNOWN AND POTENTIAL RISKS AND BENEFITS 23
6.1.5 DOSAGES, DOSAGE MODIFICATIONS, AND METHOD OF ADMINISTRATION 24
6.1.6 PREPARATION AND LABELLING 24
6.1.7 DRUG ACCOUNTABILITY 24
6.2 5-FLUOROURACIL/LEUCOVORIN 25
7. METHODS 25
7.1 OUTCOMES 25
7.1.1 PRIMARY OUTCOME 25
7.1.2 SECONDARY OUTCOMES 26
7.1.3 OTHER OUTCOMES 27
7.2 STUDY PROCEDURES 27
7.3 WITHDRAWAL OF INDIVIDUAL SUBJECTS 28
7.4 REPLACEMENT OF INDIVIDUAL SUBJECTS AFTER WITHDRAWAL 28
7.5 FOLLOW-UP OF SUBJECTS WITHDRAWN FROM TREATMENT 28
7.6 PREMATURE TERMINATION OF THE STUDY 28

8. SAFETY REPORTING 28
8.1 TEMPORARY HALT FOR REASONS OF PATIENT SAFETY 28
8.2 AEs, SAEs AND SUSARs 29
8.2.1 ADVERSE EVENTS (AEs) 29
8.2.2 SERIOUS ADVERSE EVENTS (SAEs) 29
8.2.3 SUSPECTED UNEXPECTED SERIOUS ADVERSE REACTIONS (SUSARs) 30
8.3 ANNUAL SAFETY REPORT 31
8.4 FOLLOW-UP OF ADVERSE EVENTS 31
8.5 SAFETY COMMITTEE 32
9. STATISTICAL ANALYSIS 32
10. ETHICAL CONSIDERATIONS 33
10.1 REGULATION STATEMENT 33
10.2 RECRUITMENT AND CONSENT 33
10.3 OBJECTION BY MINORS OR INCAPACITATED SUBJECTS 33
10.4 BENEFITS AND RISKS ASSESSMENT 34
10.5 COMPENSATION FOR INJURY 35
10.6 INCENTIVES 35
11. ADMINISTRATIVE ASPECTS, MONITORING AND PUBLICATION 35
11.1 HANDLING AND STORAGE OF DATA AND DOCUMENTS 35
11.2 MONITORING AND QUALITY ASSURANCE 36
11.3 AMENDMENTS 36
11.4 ANNUAL PROGRESS REPORT 36
11.5 TEMPORARY HALT AND (PREMATURELY) END OF STUDY REPORT 37
11.6 PUBLIC DISCLOSURE AND PUBLICATION POLICY 37
12. STRUCTURED RISK ANALYSIS 38
12.1 POTENTIAL ISSUES OF CONCERN 38
12.2 SYNTHESIS 38
13. REFERENCES 39
14. ATTACHMENTS 53
14.1 FIRST CHECKLIST 53
14.2 SECOND CHECKLIST 53
14.3 PROTOCOL FOR (NURSE) ANAESTHESIOLOGISTS 54
14.4 PROTOCOL FOR SCRUB NURSES 57
15. FIGURES & TABLES 66

LIST OF ABBREVIATIONS AND RELEVANT DEFINITIONS

**AE**	Adverse Event
**AR**	Adverse Reaction
**BSA**	Body-surface area
**CAWS**	Closed aerosol waste system
**CCMO**	Central Committee on Research Involving Human Subjects; in Dutch: Centrale Commissie Mensgebonden Onderzoek

**CT**	Computed tomography
**CTCAE**	Common Terminology Criteria of Adverse Events
**DW-MRI**	Diffusion weighted magnetic resonance imaging
**EudraCT**	European drug regulatory affairs Clinical Trials
**ePIPAC-OX**	Electrostatic pressurised intraperitoneal aerosol chemotherapy with oxaliplatin
**GDPR**	General Data Protection Regulation; in Dutch: Algemene Verordening Gegevensbescherming (AVG)
**IB**	Investigator's Brochure
**MCQ**	Medical Consumption Questionnaire
**METC**	Medical research ethics committee (MREC); in Dutch: medisch-ethische toetsingscommissie (METC)
**MRI**	Magnetic resonance imaging
**PCI**	Peritoneal cancer index
**PCQ**	Productivity Cost Questionnaire
**PIPAC**	Pressurised intraperitoneal aerosol chemotherapy
**PIPAC-OX**	Pressurised intraperitoneal aerosol chemotherapy with oxaliplatin
**PM**	Peritoneal metastases
**(S)AE**	(Serious) Adverse Event
**SPC**	Summary of Product Characteristics; in Dutch: officiële productinformatie IB1-tekst
**Sponsor**	The sponsor is the party that commissions the organisation or performance of the research, for example a pharmaceutical company, academic hospital, scientific organisation or investigator. A party that provides funding for a study but does not commission it is not regarded as the sponsor, but referred to as a subsidising party.
**SUSAR**	Suspected Unexpected Serious Adverse Reaction
**UAVG**	Dutch Act on Implementation of the General Data Protection Regulation; in Dutch: Uitvoeringswet AVG
**WHO**	World Health Organisation
**WMO**	Medical Research Involving Human Subjects Act; in Dutch: Wet Medisch-wetenschappelijk Onderzoek met Mensen

1

SUMMARY

Rationale: repetitive electrostatic pressurised intraperitoneal aerosol chemotherapy with oxaliplatin (ePIPAC-OX) is offered as a palliative treatment option for patients with isolated unresectable colorectal peritoneal metastases (PM) in several centres worldwide. As a palliative monotherapy, repetitive ePIPAC-OX may lead to intraperitoneal disease stabilisation with minimal treatment burden and preservation of quality of life. However, hardly anything is known about its feasibility, safety, tolerability, efficacy, costs, and pharmacokinetics in this setting.

Objectives: to prospectively explore the feasibility safety, tolerability, preliminary efficacy, costs, and pharmacokinetic profile of repetitive ePIPAC-OX as a palliative monotherapy for isolated unresectable colorectal PM under controlled circumstances.

Study design: multicentre, open-label, single-arm, phase II study.

Study population: adults who have a good performance status, adequate organ functions, histologically or cytologically confirmed unresectable isolated PM of a colorectal or appendiceal carcinoma, no symptoms of gastrointestinal obstruction, no contraindications for the planned intervention, and no previous pressurised intraperitoneal aerosol chemotherapy (PIPAC).

Intervention: instead of standard palliative treatment, enrolled patients receive laparoscopy-controlled ePIPAC-OX (92 mg/m^2 body-surface area [BSA]) with intravenous leucovorin (20 mg/m^2 BSA) and bolus 5-fluorouracil (400 mg/m^2 BSA) every six weeks. Four weeks after each procedure, patients undergo clinical, radiological, and biochemical evaluation. ePIPAC-OX is repeated until clinical, radiological, or macroscopic disease progression, after which standard palliative treatment is (re)introduced.

Main outcome: the number of patients with major toxicity (grade ≥3 according to the Common Terminology Criteria for Adverse Events v4.0) up to four weeks after the last procedure.

Burden and risks: standard palliative (systemic) treatment seems to be less effective for isolated unresectable colorectal PM compared to isolated unresectable non-peritoneal colorectal metastases. Moreover, palliative systemic therapy is associated with toxicity. As a palliative monotherapy, repetitive ePIPAC-OX may therefore lead to intraperitoneal disease stabilisation with a low toxicity, minimal treatment burden, and preservation of quality of life. If repetitive ePIPAC-OX leads to unacceptable toxicity or progression, this is detected in a sufficiently early stage by the frequent evaluations, after which standard palliative (systemic) treatment is reintroduced. Conclusively, the investigators feel that the potential benefits of participation outweigh the potential burden and risks.

1. INTRODUCTION AND RATIONALE

After the liver, the peritoneum is the second most common isolated metastatic site of colorectal cancer [1,2]. Patients with colorectal peritoneal metastases (PM) have a poor prognosis. Progression of isolated colorectal PM often lead to frequent bowel obstruction, ureteral obstruction, malignant ascites, and tumour related fatigue and cachexia. As a result, quality of life is often significantly impaired. During the last few months until death, patients often require intensive palliative care in a hospital, nursing home, hospice or in the home care setting. This translates into a significant disease burden.

The majority of patients with isolated colorectal peritoneal metastases (PM) does not qualify for curative intent surgical treatment [3], most likely due to insufficient condition or unresectable disease. Palliative systemic therapy is the standard treatment for patients with isolated unresectable colorectal PM [4]. Although its increasing use has improved the outcomes of these patients [3], palliative systemic therapy appears less effective for isolated colorectal PM compared to isolated non-peritoneal colorectal metastases [5]. This phenomenon may be explained by a relatively low intraperitoneal

concentration of systemically administered chemotherapy [6]. Moreover, a relatively high systemic concentration could cause systemic toxicity. Intraperitoneal administration of chemotherapy may increase locoregional efficacy and decrease systemic toxicity through a favourable peritoneum-plasma concentration ratio [6-8]. However, intraperitoneal chemotherapy seems to have three major limitations: a poor direct tissue penetration, an inhomogeneous intraperitoneal drug distribution, and dose-limiting local toxicity [9,10]. This has encouraged development of new intraperitoneal drug delivery systems that aim to overcome these limitations. Currently, pressurised intraperitoneal aerosol chemotherapy (PIPAC) is one of these systems that internationally gains the most attention [11].

1.1 PIPAC

PIPAC is a laparoscopy-controlled repetitive intraperitoneal administration of low-dose chemotherapy as a pressurised aerosol [11-13]. It combines the theoretical pharmacokinetic advantages of low-dose intraperitoneal chemotherapy (i.e. low toxicity, high intraperitoneal concentration, low systemic concentration) with the principles of an aerosol (homogeneous intraperitoneal distribution) and intra-abdominal pressure (deep tissue penetration) [12-20]. Two research groups systematically reviewed results of non-comparative clinical studies that investigated the feasibility, safety, tolerability, and preliminary efficacy of PIPAC with various drugs for PM of various origins [21,22]. They concluded that PIPAC appears feasible, safe, and well tolerated, may stabilise quality of life, and could induce cytological and histological regression of PM [21,22]. These preliminary conclusions have led to an increasing acceptance of PIPAC as a palliative treatment option for PM in several centres worldwide [23]. In these centres, patients with isolated unresectable colorectal PM usually receive PIPAC with oxaliplatin (PIPAC-OX) in an empirically chosen dosage of 92 mg/m^2 body-surface area (BSA) every four to six weeks [23]. Some centres use electrostatic precipitation of the aerosol during PIPAC-OX (ePIPAC-OX) [24,25], since this could increase tissue penetration of oxaliplatin [26].

1.2 PIPAC FOR COLORECTAL PM

Several clinical studies included patients who received repetitive PIPAC-OX for colorectal PM [27-36]. However, the vast majority of these studies reported outcomes of entire cohorts that received repetitive PIPAC with various drugs for PM of various origins without presenting subgroup analyses of patients who received PIPAC-OX for colorectal PM [27-34]. Only two studies reported separate outcomes of repetitive PIPAC-OX for colorectal PM [35,36]. By using a prospectively maintained database, Teixeira-Farinha *et al* retrospectively included 20 patients with isolated colorectal PM who received 37 procedures [35]. They concluded that repetitive PIPAC-OX causes a modest and transitory inflammatory response without haematological, renal, or hepatic toxicity [35]. Demtröder *et al.* retrospectively included 17 patients with isolated colorectal PM who received 48 procedures within an off-label program [36]. They concluded that repetitive PIPAC-OX induces regression of pretreated colorectal PM and that the toxicity seems to be low [36]. Both studies have a retrospective design, no predefined population, and no prespecified endpoints. Moreover, both studies included patients who receive repetitive PIPAC-OX as a monotherapy as well as patients who receive PIPAC-OX in combination with palliative systemic therapy. These shortcomings strongly impede the interpretation of these studies. Besides, recently published case reports suggested that PIPAC-OX could lead to severe hypersensitivity reactions and peritoneal sclerosis [37,38].

1.3 RATIONALE FOR THIS STUDY

Conclusively, little is known about the safety, tolerability, and efficacy of repetitive PIPAC-OX in patients with isolated unresectable colorectal PM, whereas nothing is known about its costs and pharmacokinetic profile. Specifically for repetitive ePIPAC-OX, all these outcomes have never been reported. This questions the current use of repetitive (e)PIPAC-OX as a palliative treatment option for isolated unresectable colorectal PM outside the framework of clinical study protocols. Ideally, these patients are included in prospective studies with prespecified eligibility criteria, interventions, and endpoints. However, by the knowledge of the investigators, such studies are currently lacking and not

ongoing [39]. Therefore, this study aims to prospectively explore the safety, tolerability, preliminary efficacy, costs, and pharmacokinetics of repetitive ePIPAC-OX as a palliative therapy for isolated unresectable colorectal PM. Although implementation of PIPAC appears feasible and occupationally safe [21,22,24,40-43], there is no experience with PIPAC in the Netherlands. Hence, this study also aims to assess the feasibility of implementation of ePIPAC-OX in two Dutch tertiary referral hospitals for the surgical treatment of colorectal PM.

1.4 RATIONALE FOR INTERVENTION

Repetitive ePIPAC-OX may be administered as part of a bidirectional therapy with palliative systemic therapy or as a monotherapy. When administered as a bidirectional therapy, the main objective is maximising tumour response, probably at the expense of an increased treatment burden that could interfere with quality of life. When administered as a monotherapy, the main objective is temporary intraperitoneal disease stabilisation with minimal treatment burden and preservation of quality of life. For this study, the investigators decided to administer repetitive ePIPAC-OX as a palliative monotherapy under controlled circumstances, with (re)introduction of standard palliative treatment upon progression. According to internationally used protocols, ePIPAC-OX is administered in a dosage of 92 mg/m^2 at six-weekly intervals [23]. Before administration of ePIPAC-OX, patients receive intravenous low-dose leucovorin with bolus 5-fluorouracil, since this is thought to potentiate the effect of intraperitoneal oxaliplatin [44,45].

2. OBJECTIVES

2.1 PRIMARY OBJECTIVE

The primary objective of this study is to explore the safety of repetitive ePIPAC-OX as a palliative monotherapy for patients with isolated unresectable colorectal PM.

2.2 SECONDARY OBJECTIVES

Secondary objectives of this study are to explore the tolerability, preliminary efficacy, costs, and pharmacokinetics of repetitive ePIPAC-OX as a palliative monotherapy for patients with isolated unresectable colorectal PM. Although implementation of PIPAC appears feasible and occupationally safe [21,22,24,40-43], there is no experience with PIPAC in the Netherlands. Hence, this study also aims to assess the feasibility of implementation of ePIPAC-OX in two Dutch tertiary referral hospitals for the surgical treatment of colorectal PM. Lastly, this study aims to systematically collect blood and tissue from enrolled patients for future translational research.

3. STUDY DESIGN

3.1 DESIGN

This is a prospective, open-label, single-arm, phase II study. *Figure 1* shows a general flowchart of the study. *Table 1* presents a schedule of enrolment, interventions, and assessments (section 15).

3.2 SETTING

This study is performed in two Dutch teaching hospitals qualified as tertiary referral hospitals for the surgical treatment of colorectal PM. Both study centres are qualified for PIPAC.

3.3 DURATION

The investigators expect that a sufficient number of patients are referred to the study centres, since both study centres are actively involved in the nationwide Dutch Peritoneal Oncology Group. Therefore, the investigators anticipate that 20 patients are enrolled within two years.

4. STUDY POPULATION

4.1 POPULATION (BASE)

In the Netherlands, ±25% of patients with synchronous metastases of colorectal cancer have PM [46]. Of these patients with colorectal PM, ±50% presents with 'isolated colorectal PM' without radiological evidence of systemic metastases [46]. This results in ±375 patients with isolated synchronous colorectal PM in the Netherlands each year (www.cijfersoverkanker.nl). The incidence of metachronous colorectal PM is also known to be relevant [47]. However, exact numbers in the Netherlands are difficult to determine, since metachronous metastases are not registered in the Netherlands Cancer Registry. Based on clinical experience, the investigators expected that another ±375 patients are diagnosed with isolated metachronous colorectal PM each year. This results in an estimated total of ±750 patients with isolated colorectal PM in the Netherlands each year, of whom ±525 patients do not qualify for curative intent treatment [3], frequently because of unresectable disease. These ±525 patients with isolated unresectable colorectal PM are potential study candidates.

4.2 ELIGIBILITY CRITERIA

Eligible patients are adults who have:

- a World Health Organisation (WHO) performance status of ≤1 and life expectancy >3 months;
- histological or cytological proof of PM of a colorectal or appendiceal carcinoma;
- unresectable disease determined by abdominal computed tomography (CT) and a diagnostic laparoscopy or laparotomy;
- adequate organ functions (haemoglobin ≥5.0 mmol/L, neutrophils ≥1.5 x 10^9/L, platelets ≥100 x 10^9/L, serum creatinine <1.5 x ULN, creatinine clearance ≥30 ml/min, and liver transaminsases <5 x ULN);
- no symptoms of gastrointestinal obstruction;
- no radiological evidence of systemic metastases;

- no contraindications for oxaliplatin or 5-fluorouracil/leucovorin;
- no contraindications for a laparoscopy;
- no previous PIPAC-procedures;
- written informed consent.

Importantly, enrolment is allowed for patients with an unresected primary tumour (if asymptomatic) and for patients in various lines of palliative treatment, including patients who refuse, have not had, or do not qualify for first-line palliative systemic therapy. All potentially eligible patients are discussed in a multidisplinary team. Enrolled patients need to be informed about the potential consequences of postponing or discontinuing standard palliative treatment by a medical oncologist prior to enrolment.

4.3 SAMPLE SIZE

The sample size had to be determined pragmatically rather than statistically. As PIPAC is not insured in the Netherlands, all procedures need to be funded by the investigators. The awarded grant enables funding of 60 procedures. Since the expected mean number of procedures is three per patient [36], the sample size is determined at 20 patients. The sample size may, if approved by the METC, be enlarged if more funding becomes available or reduced if the mean number of procedures per patient exceeds three.

5. TREATMENT OF SUBJECTS

Figure 1 shows a flow chart of the study. *Table 1* presents a schedule of enrolment, interventions, and assessments.

5.1 ePIPAC-OX

The procedure-related principles of (e)PIPAC have been extensively described by Willaert *et al* and Giger-Pabst *et al* [24,48]. In this study, ePIPAC-OX is performed at six-weekly intervals by at least one

PIPAC-qualified surgeon in a standard operating room with laminar airflow. Procedures are performed under general anaesthesia without antibiotic prophylaxis or venous thromboembolism prophylaxis. Before each procedure, a checklist (section 14.1) is used to ensure all materials are available. The operating personnel wears appropriate chemotherapy-protective clothes according to existing HIPEC protocols. Section 14 provides Dutch procedure-specific protocols developed by anaesthesiologists (section 14.3) and scrub nurses (section 14.4).

The Hasson technique is used to insert a 10 mm blunt tip balloon trocar through the abdominal wall. After obtaining a normothermic 12 mmHg capnoperitoneum, a second 10 mm blunt tip balloon trocar is inserted under direct vision and explorative laparoscopy is performed. Only if needed, careful adhesiolysis may be performed to create sufficient working space. In case of an iatrogenic bowel laesion, the procedure is ended after closure of the laesion, and ePIPAC-OX may be postponed by two to four weeks. If the procedure is considered feasible, leucovorin (20 mg/m^2 BSA in 10 minutes) and bolus 5-fluorouracil (400 mg/m^2 BSA in 15 minutes) are administered intravenously. In the meantime, ascites (or injected saline if ascites is not present) is completely evacuated, sent for cytology and translational research, and the ascites volume is documented. The Zühlke score and the peritoneal cancer index (PCI) are registered and photographs are taken throughout the peritoneal cavity [49,50]. Three peritoneal metastases, preferably from different areas, are biopsied, sent for histopathology and translational research, and their locations are documented and marked with clips to enable biopsies of the same locations during subsequent procedures.

Then, the ePIPAC installation is build. A stainless steel brush electrode (Ionwand®, Alesi Surgical, Cardiff, United Kingdom) is inserted through a mini-trocar under direct vision, secured with its tip at least 2 cm away from other structures, and connected to its generator (Ultravision®, Alesi Surgical, Cardiff, United Kingdom). An aerosoliser (CapnoPen®, Capnomed GmbH, Villingendorf, Germany) is inserted through one of the trocars and secured with its nozzle just inside the peritoneal cavity at a reasonable distance from visceral organs. The camera, inserted through the other trocar, is secured by a laparoscope holder in a way it permanently visualises the electrode and the aerosoliser. The valve of

the trocar connected to the CO_2 insufflation remains opened, whereas the other trocar is connected to a closed aerosol waste system (CAWS) with its valve closed. The CAWS consecutively consists of a smoke evacuation filter, a water seal drainage system, an infant-paediatric electrostatic microparticle filter, and the air waste system of the hospital. The preoperatively prepared syringe with oxaliplatin (92 mg/m^2 BSA diluted in 150 ml 5% dextrose) is vented, placed in an angiographic injector, and connected to the aerosoliser with a saline-flushed high-pressure line protected by a plastic camera cover. A leak-free capnoperitoneum is ensured by zero-flow of CO_2. If necessary, incisions may be additionally sutured and Luer lock caps may be placed on balloon valves of trocars. The angiographic injector is installed at a flow rate of 30 ml/min and a maximum pressure of 200 pounds per square inch. Protective films are placed on the floor below the angiographic injector and around the patient. The angiographic injector is positioned above a chemotherapy waste bin. The peripheral venous line of the patient is connected to a 60 ml saline-containing syringe outside the operating room. Vital parameters of the patient, real-time videolaparoscopy, and a patient-aimed camera are displayed on three screens outside the operating room. The screen of the angiographic injector is positioned in front of the window of the operating room. General anaesthesia is ensured for at least another 40 minutes. A checklist is used to confirm that all aforementioned steps have been adequately taken. After completion of the checklist (section 14.2), the entire operating personnel leaves the operating room.

Oxaliplatin is injected through the aerosoliser by remote controlled activation of the angiographic injector from outside the operating room. After complete formation of the oxaliplatin-containing aerosol in 5 minutes, the surgeon enters the operating room and turns on the Ultravision® generator, after which the aerosol is electrostatically precipitated. The electrostatic field and the capnoperitoneum are maintained for another 25 minutes. During this phase, the patient and the procedure are monitored through the three screens and the window of the operating room. Drugs may be administered through the intravenous access outside the operating room if necessary.

After 25 minutes, the surgeon enters the operating room, turns off the Ultravision® generator, closes the trocar valve connected to the CO_2 insoufflation, and opens the trocar valve connected to

the CAWS. After complete evacuation of the aerosol, the electrode and the aerosoliser are removed, the entire operating personnel enters the operating room, and a new capnoperitoneum is obtained. Ascites and peritoneal biopsies are collected for pharmacokinetic purposes. In case no bleeding or perforations are observed, instruments are removed and incisions are closed with absorbable sutures. All instruments and materials are directly disposed in chemotherapy waste bins and the operating room is cleaned according to existing HIPEC protocols.

After ePIPAC-OX, patients are admitted to the general surgical ward. To relieve postoperative pain, patients receive paracetamol (1 g, four times daily), on-demand morphine, and 1 g of metamizole directly after the procedure. To minimise postoperative nausea and vomiting, patients receive perioperative dexamethasone and on-demand granisetron (1 mg, three times daily). Standard post-surgical clinical evaluations are performed a few hours after the procedure and on every postoperative day. Blood is drawn for bone marrow, liver, and kidney functions, albumin, and C-reactive protein on every postoperative day. If the postoperative period is uneventful, patients are discharged on the first postoperative day. All body excretes are considered oxaliplatin-contaminated for up to five days after the procedure.

Dose reduction, prohibited and permitted concomitant care, and strategies to improve adherence are not specified *a priori*, but left to the discretion of the treating physician. ePIPAC-OX is repeated until clinical, radiological, or macroscopic (i.e. ascites, PCI) progression, unacceptable toxicity, physicians decision to discontinue, or at patients request to discontinue. In patients who develop systemic metastases, continuation of ePIPAC-OX can only be considered if the patient has no systemic palliative treatment options and stable peritoneal disease.

5.2 OUTPATIENT EVALUATIONS

One week after each ePIPAC-OX, patients undergo clinical evaluation by phone. Four weeks after each ePIPAC-OX, patients undergo radiological evaluation (i.e. thoracoabdominal CT, diffusion-weighted

magnetic resonance imaging [DW-MRI]), biochemical evaluation (i.e. bone marrow, liver, and kidney functions, albumin, C-reactive protein, tumour markers), and clinical evaluation.

5.3 QUESTIONNAIRES

Patients are asked to complete EQ-5D-5L, QLQ-C30, and QLQ-CR29 at baseline and one and four weeks after each ePIPAC-OX [51-53]. iMTA Productivity Cost Questionnaire (PCQ) and iMTA Medical Consumption Questionnaire (MCQ) are sent to the patients at baseline and four weeks after each ePIPAC-OX (PCQ) and each second ePIPAC-OX (MCQ) [54,55]. According their own preference, patients receive questionnaires either by mail or digitally by using an ISO 27001 certified data management system (De Research Manager, Deventer, Netherlands).

5.4 PHARMACOKINETICS

Blood is collected during and after the first three procedures in each patient. Four ml of blood is drawn and collected in heparin tubes before ePIPAC-OX and 5, 10, 20, 30, 60, 120, 240, 360, and 1080 minutes after injection of oxaliplatin from a thick peripheral intravenous catheter that is placed after general anaesthesia during ePIPAC-OX. This means no additional venapunctions for the patient. After immediate centrifuging, an aliquot of plasma is stored at -80°C until analysis and another aliquot of plasma is centrifuged through an ultrafiltration membrane and stored at -80°C until analysis. Urine, ascites, peritoneal metastases, and normal peritoneum are collected during and after all procedures. Four ml of urine is collected in urinalysis tubes before ePIPAC-OX and on the first postoperative day. These are stored at -20°C until analysis. After discharge, patients are asked to collect 10 ml of urine in urinalysis tubes on the third, fifth, and seventh postoperative day, and to store these specimens at their home address at -20°C until analysis. After electrostatic precipitation of the aerosol, the surgeon aspirates a few ml of ascites and biopsies two peritoneal metastases and two pieces of normal peritoneum, preferably from different locations. These are collected in aliquots and directly stored at

-80°C until analysis. Concentrations of oxaliplatin are measured by atomic absorption spectrophotometry after completion of the study.

5.5 TRANSLATIONAL RESEARCH

Before each ePIPAC-OX, 20 ml of blood is drawn and collected in 10 ml Cell-free DNA BCT tubes (Streck, La Vista, NE, USA) from an intravenous catheter that is already in situ for ePIPAC-OX. This means no additional venapunction for the patient. According to the manufacturer's instructions, these tubes are sent to a central lab for isolation and storage (-80°C) of plasma and cell pellet. A part of each biopsied PM for histopathology (section 5.1) and collected ascites for cytology (section 5.1) is separately processed and stored for translational research.

6. INVESTIGATIONAL PRODUCT

6.1 OXALIPLATIN

6.1.1 NAME AND DESCRIPTION

The first investigational product is oxaliplatin (L01XA03) in a dosage of 92 mg/m^2 BSA (max 2 m^2 BSA), diluted in 150 ml 5% glucose, administered intraperitoneally at six-weekly intervals. Oxaliplatin has a marketing authorisation for the treatment of metastatic colorectal cancer. The Summary of Product Characteristics of oxaliplatin can be found here.

6.1.2 SUMMARY OF FINDINGS OF NON-CLINICAL STUDIES

Given the extensive experience with intraperitoneal and systemic oxaliplatin in human beings, non-clinical studies are not discussed. The Summary of Product Characteristics of oxaliplatin can be found here.

6.1.3 SUMMARY OF FINDINGS OF CLINICAL STUDIES

Between 2002 and 2017, the medical community has gained extensive clinical experience with high-dose (300-460 mg/m^2 BSA) intraperitoneal oxaliplatin after cytoreductive surgery in the treatment of PM [56-117]. The locoregional intraperitoneal toxicity (e.g. bowel perforation, intraperitoneal haemorrhage, paralytic ileus) of oxaliplatin after cytoreductive surgery is difficult to determine in the studies, since it may be strongly influenced by cytoreductive surgery. Nevertheless, based on these clinical and pharmacological studies, intraperitoneal oxaliplatin in a dosage of 300-460 mg/m^2 BSA is currently accepted as a standard drug after cytoreductive surgery in many countries worldwide, including the Netherlands [4]. Moreover, it is used in dosages up to 460 mg/m^2 in several ongoing trials such as the Belgian BEV-IP trial and the Dutch COLOPEC trial, which also runs in both study centres [118,119]. Importantly, CRC-PIPAC uses intraperitoneal oxaliplatin in a dosage of 92 mg/m^2, which is considerably lower than the dosage used after cytoreductive surgery. Moreover, the dosage of 92 mg/m^2 is used in ongoing Italian and Danish trials focusing on PIPAC-OX for colorectal PM (NCT02504784, NCT03287375).

In 17 patients who underwent repetitive PIPAC-OX (92 mg/m^2), Demtröder *et al* showed no intraoperative complications, and no major (CTCAE grade ≥4) locoregional intraperitoneal toxicity after single or repeated PIPAC-OX. CTCAE grade 3 locoregional intraperitoneal toxicity was observed in four patients (23%), the most frequent being abdominal pain [36]. In summary, the locoregional intraperitoneal toxicity of PIPAC-OX (92 mg/m^2) is thought to be low, especially lower than the locoregional intraperitoneal toxicity of oxaliplatin (300-460 mg/m^2) used after cytoreductive surgery.

The standard treatment of patients with unresectable metastatic colorectal cancer (population in this study) includes intravenous oxaliplatin in a dosage of 85 mg/m^2 every 2 weeks or 130 mg/m^2 every 3 weeks [4]. Risks and side effects associated with this dose are extensively described in the Summary of Product Characteristics. The investigators expect that the systemic toxicity of repetitive ePIPAC-OX (92 mg/m^2) every 6 weeks is much lower than the intravenous dose of 85-130 mg/m^2 every two to three weeks. This expectation was confirmed by Demtröder et al., who showed only minimal liver

and/or renal toxicity of CTCAE grade 1 in six of seventeen patients who were treated with repetitive PIPAC-OX (92 mg/m^2) in combination with systemic therapy [36]. Since patients in this study are treated with repetitive PIPAC-OX (92 mg/m^2) as a monotherapy, the investigators hypothesise that the risk of systemic toxicity in this study is very low. Patients not participating in the study are intentionally treated with palliative systemic therapy, and therefore exposed to the potentially higher risk of systemic toxicity of intravenous oxaliplatin.

6.1.4 SUMMARY OF KNOWN AND POTENTIAL RISKS AND BENEFITS

The most important potential risks of PIPAC-OX are locoregional and systemic toxicity. Locoregional toxicity may include bowel perforation, intraperitoneal haemorrhage, paralytic ileus, nausea/vomiting, and abdominal pain. However, in 17 patients, Demtröder *et al* did not observe any bowel perforation or intraperitoneal haemorrhage, whereas nausea and vomiting and/or abdominal pain were observed in 4 patients with a CTCAE grade 3 [36]. Therefore, the risk of major locoregional toxicity appears to be low. Systemic toxicity may include peripheral sensory neuropathy, neutropenia, anaemia, thrombocytopenia, leukopenia, nausea, vomiting, diarrhoea, constipation, abdominal pain, mucositis, stomatitis, fever, fatigue, asthenia, alopecia, infections, anorexia, loss of taste, anaphylactic reactions, headache, back pain, dyspnoea, cough, epistaxis, weight changes, glycaemic disorders, electrolyte disorders, and liver function disorders (www.farmacotherapeutischkompas.nl). However, given the low expected systemic uptake, the investigators hypothesise that PIPAC-OX (92 mg/m^2) every six weeks carries a low risk of systemic toxicity, especially when compared to the standard intravenous dose of 85-130 mg/m^2 every two to three weeks. Potential benefits of PIPAC-OX are tumour stabilisation or regression with minimal treatment burden and preservation of quality of life [21,22,36].

6.1.5 DOSAGES, DOSAGE MODIFICATIONS, AND METHOD OF ADMINISTRATION

The dosage of ePIPAC-OX (92 mg/m^2 diluted in 150 ml of 5% dextrose) is based on previous literature, currently ongoing studies, and recommendations of the most experienced PIPAC-centres worldwide [23]. No dosage modifications are defined *a priori*. At the moment, there are two ongoing phase I dose escalation studies for PIPAC-OX (NCT03294252, NCT03172416). We will frequently contact them to evaluate whether they recommend another dosage based on the findings of their study. If so, we will discuss this potential dosage modification with the METC.

6.1.6 PREPARATION AND LABELLING

A 5 mg/ml solution of oxaliplatin at a dose of 92 mg/m^2 BSA is transferred into a 150 ml syringe compatible with the angiographic injector. This solution is filled with a 5% (50 mg/ml) dextrose solution up to a total of 150 ml. Available literature in the *handbook on injectable drugs* of the American Society for Health-system Pharmacists indicates that oxaliplatin remains stable in a 5% dextrose solution at room temperature, with a degradation of <4% after 90 days. Based on this literature, the study centres store oxaliplatin in 5% glucose at room temperature and consider the solution to be sustainable until 7 days after preparation.

6.1.7 DRUG ACCOUNTABILITY

Oxaliplatin is a registered drug for systemic administration in patients with colorectal cancer [4], and HIPEC with oxaliplatin is standard of care in HIPEC centres in the Netherlands [4], including our own hospital. Given the extensive experience with oxaliplatin, the medication for this study is used from commercial stock and preparation and labelling are performed on a patient-named basis within the hospital pharmacy department. Therefore, no specific labelling for research purposes is performed in this study.

6.2 5-FLUOROURACIL/LEUCOVORIN

The second and third investigational products are bolus 5-fluorouracil (L01BC02) and low-dose leucovorin (V03AF03), administered intravenously in a dose of 400 mg/m^2 and 20 mg/m^2, respectively. In the Netherlands, 5-fluorouracil and leucovorin have had a marketing authorisation for the treatment of colorectal cancer for decades. Their Summary of Product Characteristics can be found here (5-fluorouracil) and here (leucovorin). For several reasons, the investigators feel it is not necessary to provide a summary of (non-)clinical studies, a summary of risks and benefits, or detailed information about dosage, dosage modifications, method of administration, preparation, labelling, and/or drug accountability. Firstly, there is extensive experience with the intravenous administration of the combination of these drugs for the indication in this study (metastatic colorectal cancer). Secondly, the dosage used in this study is lower than the standard systemic dosage during palliative systemic therapy. Thirdly, both study centres have extensive experience with administering 5-fluorouracil/leucovorin in these dosages during HIPEC with oxaliplatin [4]. The medication is used from commercial stock and preparation and labelling are performed on a patient-named basis within the hospital pharmacy department according to existing protocols.

7. METHODS

An assessment schedule is presented in *Table 1*. The primary outcome is the number of patients with major toxicity, defined as grade ≥3 according to the Common Terminology Criteria for Adverse Events (CTCAE) v4.0 [120], up to four weeks after the last ePIPAC-OX.

7.1 OUTCOMES

7.1.1 PRIMARY OUTCOME

The primary outcome is the number of patients with major toxicity, defined as grade ≥3 according to the Common Terminology Criteria for Adverse Events (CTCAE) v4.0 [120], up to four weeks after the last ePIPAC-OX.

7.1.2 SECONDARY OUTCOMES

Secondary outcomes are:

- the environmental safety of ePIPAC-OX, based on air concentrations (measured by RPS Analyse, Breda, Netherlands) and surface concentrations (measured by Pharmacy, Catharina Hospital, Eindhoven, Netherlands) of oxaliplatin during the first three procedures, measured by atomic absorption spectrophotometry;
- procedure-related characteristics of ePIPAC-OX (e.g. laparoscopic access, intraoperative complications, amount of adhesions, technical difficulties, operating time);
- the number of procedures in each patient and reasons for discontinuation;
- minor toxicity, defined as grade ≤2 according to CTCAE v4.0 [120], up to four weeks after the last ePIPAC-OX;
- organ-specific toxicity, based on bone marrow, liver, and kidney functions measured at different time points (Table 1);
- major and minor postoperative complications, defined as grade ≥3 and grade ≤2 according to Clavien-Dindo [121], respectively, up to four weeks after the last ePIPAC-OX;
- hospital stay, defined as the number of days between ePIPAC-OX and initial discharge;
- readmissions, defined as any hospital admission after initial discharge, up to four weeks after the last ePIPAC-OX;
- radiological tumour response, based on central review of thoracoabdominal CT and DW-MRI at baseline and four weeks after each ePIPAC-OX, performed by two independent radiologists (JN, MLH) blinded to clinical outcomes (classification is not defined *a priori*);
- histopathological tumour response, based on central review of collected peritoneal biopsies during each ePIPAC-OX, performed by two independent pathologists (e.g. CJRH) blinded to clinical outcomes by using the Peritoneal Regression Grading Score [122];

- cytological tumour response, based on collected ascites or peritoneal washing cytology during each ePIPAC-OX;
- macroscopic tumour response, based on PCI and ascites volume during each ePIPAC-OX;
- biochemical tumour response, based on tumour markers measured at different time points (Table 1);
- quality of life, extracted from questionnaires (EQ-5D-5L, QLQ-C30, QLQ-CR29) at different time points (Table 1);
- costs, derived from the Dutch costing guidelines for health care research at the time of analysis, based on case report forms, hospital information systems, and questionnaires (iMTA PCQ, iMTA MCQ) at different time points (Table 1);
- progression-free survival, defined as the time between enrolment and clinical, radiological, or macroscopic progression, or death;
- overall survival, defined as the time between enrolment and death.

7.1.3 OTHER OUTCOMES

In each patient, baseline parameters are registered after enrolment (gender, age, date of diagnosis with colorectal cancer, date of diagnosis with colorectal PM, metachronous/synchronous, previous surgical and systemic treatments, WHO performance status, primary tumour location, histology, T-stage, N-stage, and primary tumour differentiation, etcetera).

7.2 STUDY PROCEDURES

Figure 1 shows a general flowchart of the study. *Table 1* presents a detailed schedule of enrolment, interventions, and assessments.

7.3 WITHDRAWAL OF INDIVIDUAL SUBJECTS

Subjects can leave the study at any time for any reason if they wish to do so without any consequences. The investigator can decide to withdraw a subject from the study for urgent medical reasons.

7.4 REPLACEMENT OF INDIVIDUAL SUBJECTS AFTER WITHDRAWAL

Enrolled patients who do not undergo a first ePIPAC-OX (e.g. systemic metastases on baseline radiology, non-access, resectable disease) are replaced to enrol 20 patients who receive at least one ePIPAC-OX.

7.5 FOLLOW-UP OF SUBJECTS WITHDRAWN FROM TREATMENT

Outcomes are collected and followed in all patients who receive at least one ePIPAC-OX.

7.6 PREMATURE TERMINATION OF THE STUDY

Interim analyses are performed after 8 and 20 procedures. The study is terminated after these interim analyses if CTCAE grade ≥3 toxicity, directly related to ePIPAC-OX, is observed after ≥4 and ≥10 procedures. Furthermore, the study is directly terminated if more than one CTCAE grade 5 toxicity, directly related to ePIPAC-OX, occurs during the study. The coordinating investigator and the principal investigator have access to these interim results. The principal investigator makes the decision to terminate or continue the study.

8. SAFETY REPORTING

8.1 TEMPORARY HALT FOR REASONS OF PATIENT SAFETY

In accordance to section 10, subsection 4, of the WMO, the sponsor suspends the study if there is sufficient ground that continuation of the study jeopardises patient health or safety. The sponsor notifies the accredited METC without undue delay of a temporary halt including the reason for such

an action. The study is suspended pending a further positive decision by the accredited METC. The coordinating investigator takes care that all subjects are kept informed.

8.2 AEs, SAEs AND SUSARs

8.2.1 ADVERSE EVENTS (AEs)

Adverse events are defined as any undesirable experience occurring to a patient during the study, whether or not considered related to study participation. All adverse events reported spontaneously by the patient or observed by the investigators are recorded.

8.2.2 SERIOUS ADVERSE EVENTS (SAEs)

A serious adverse event is any untoward medical occurrence or effect that:

- results in death;
- is life threatening (at the time of the event);
- requires hospitalisation or prolongation of existing inpatients' hospitalisation;
- results in persistent or significant disability or incapacity;
- is a congenital anomaly or birth defect; or
- any other important medical event that did not result in any of the outcomes listed above due to medical or surgical intervention but could have been based upon appropriate judgement by the investigator.

An elective hospital admission is not considered as a serious adverse events. The investigators report all SAEs to the coordinating investigator without undue delay after obtaining knowledge of the events. The coordinating investigator report the SAEs through the web portal *ToetsingOnline* to the accredited METC that approved the protocol, within 7 days of first knowledge for SAEs that result in death or are

life threatening followed by a period of maximum of 8 days to complete the initial preliminary report. All other SAEs are reported within a period of maximum 15 days after the sponsor has first knowledge of the serious adverse events.

8.2.3 SUSPECTED UNEXPECTED SERIOUS ADVERSE REACTIONS (SUSARs)

Adverse reactions are all untoward and unintended responses to an investigational product related to any dose administered. Unexpected adverse reactions are SUSARs if the following three conditions are met:

- the event must be serious (see chapter 9.2.2);
- there must be a certain degree of probability that the event is a harmful and an undesirable reaction to the medicinal product under investigation, regardless of the administered dose;
- the adverse reaction must be unexpected, that is to say, the nature and severity of the adverse reaction are not in agreement with the product information as recorded in:
  - Summary of Product Characteristics (SPC) for an authorised medicinal product;
  - Investigator’s Brochure for an unauthorised medicinal product.

The coordinating investigator reports expedited the following SUSARs through the web portal *ToetsingOnline* to the METC:

- SUSARs that have arisen in the clinical trial that was assessed by the METC;
- SUSARs that have arisen in other clinical trials of the same sponsor and with the same medicinal product, and that could have consequences for the safety of the subjects involved in the clinical trial that was assessed by the METC.

The remaining SUSARs are recorded in an overview list (line-listing) that is submitted once every half year to the METC. This line-listing provides an overview of all SUSARs from the study medicine, accompanied by a brief report highlighting the main points of concern. The expedited reporting of SUSARs through the web portal Eudravigilance or ToetsingOnline is sufficient as notification to the competent authority. The coordinating investigator reports expedited all SUSARs to the competent authorities in other Member States, according to the requirements of the Member States. The expedited reporting occurs not later than 15 days after the coordinating investigator has first knowledge of the adverse reactions. For fatal or life threatening cases the term is maximal 7 days for a preliminary report with another 8 days for completion of the report.

8.3 ANNUAL SAFETY REPORT

In addition to the expedited reporting of SUSARs, the sponsor submits, once a year throughout the clinical trial, a safety report to the accredited METC, competent authority, and competent authorities of the concerned Member States. This safety report consists of:

- a list of all suspected (unexpected or expected) serious adverse reactions, along with an aggregated summary table of all reported serious adverse reactions, ordered by organ system, per study;
- a report concerning the safety of the subjects, consisting of a complete safety analysis and an evaluation of the balance between the efficacy and the harmfulness of the medicine under investigation.

8.4 FOLLOW-UP OF ADVERSE EVENTS

All AEs are followed until they have abated, or until a stable situation has been reached. Depending on the event, follow up may require additional tests or medical procedures as indicated, and/or referral

to the general physician or a medical specialist. SAEs need to be reported until four weeks after the last ePIPAC-OX.

8.5 SAFETY COMMITTEE

The investigators decided that a data monitoring committee is not needed given the clear stopping rules and the low expected toxicity of repetitive ePIPAC-OX [21,22,36]. However, there may be some need for external safety review. Four weeks after the 8th and 20th PIPAC procedure, the toxicity results are evaluated by the investigators. If the investigators doubt about whether to continue the study based on these results, they may send the results out for feedback to an external safety committee. This committee consists of dr. Pieter J. Tanis (surgeon, Academic Medical Centre, Amsterdam, Netherlands) and prof. dr. C.J.A. Punt (medical oncologist, Academic Medical Centre, Amsterdam, Netherlands). Both physicians are independent of the members, since they are no employees of the study centres, and since the Academic Medical Centre is no tertiary referral centre for the surgical treatment of colorectal PM. This committee may provide additional recommendations to the investigators. The decision to continue or terminate the study remains with the investigators.

9. STATISTICAL ANALYSIS

Categorical variables are presented as *n* (%). Continuous variables are presented as mean (standard deviation) or median (range) where appropriate. Repetitive continuous outcomes (e.g. organ toxicity, quality of life, operating time) are analysed by using the Wilcoxon signed-rank test, the paired samples t-test, the Friedman test, or repeated measurements analysis of variance where appropriate. Repetitive categorical outcomes (e.g. intraoperative complications, postoperative complications) are analysed by using the McNemar test, the Wilcoxon signed-rank test, the Cochran’s Q test, or generalised estimating equations where appropriate. Time-to-event variables (i.e. overall and progression-free survival) are analysed and displayed by using the Kaplan-Meier method. All other outcomes, including the interim analyses (section 7.6), are analysed by using descriptive statistics.

Statistical methods for pharmacokinetic analyses are not specified *a priori.* All statistical tests are two-sided and p <0.05 is considered statistically significant.

10. ETHICAL CONSIDERATIONS

10.1 REGULATION STATEMENT

The study is conducted according to the principles of the Declaration of Helsinki (see www.wma.net for the most recent version) and in accordance with the Medical Research Involving Human Subjects Act (WMO).

10.2 RECRUITMENT AND CONSENT

Informed consent is obtained by local investigators at the outpatient clinics of the study centres. The investigators expect that the vast majority of patients are referred to the study centres specifically for this study. Firstly, patients are briefly informed about the study by a local investigator, after which the patient information sheet is provided. Thereafter, patients mostly enter a period of investigations (bloods, laparoscopy, scan) during which they have sufficient time to consider their decision. The patient information letter contains contact details of the study team in case there are additional questions. After the period of investigations, patients are seen on the outpatient clinics for results of the investigations and the decision to (not) participate in the study. Patients are given the possibility to give separate permission for undergoing DW-MRI and for storage of specimens for translational research.

10.3 OBJECTION BY MINORS OR INCAPACITATED SUBJECTS

No minors or incapacitated subjects are included.

10.4 BENEFITS AND RISKS ASSESSMENT

The first risk associated with participation is locoregional toxicity of repetitive ePIPAC-OX. However, the locoregional toxicity of PIPAC-OX appears to be low [36]. Furthermore, the dosage of ePIPAC-OX (oxaliplatin 92 mg/m^2 BSA) is much lower than the widely accepted dose of oxaliplatin during HIPEC (300-460 mg/m^2 BSA), which is also used in the COLOPEC trial in both study centres.

The second risk associated with participation is systemic toxicity of repetitive ePIPAC-OX. However, the systemic toxicity of repetitive ePIPAC-OX (92 mg/m^2 BSA every six weeks) is likely to be considerably lower than the systemic toxicity of oxaliplatin during palliative systemic therapy (80-130 mg/m^2 BSA every two to three weeks). When repetitive ePIPAC-OX eventually turns out to be too toxic, this is detected by the clearly defined interim analyses and stopping rules (section 7.6). Finally, patients with contraindications for the planned chemotherapy (e.g. previous allergic reaction) cannot be included in the study.

The last risk is inefficacy of repetitive ePIPAC-OX. However, preliminary results of repetitive ePIPAC-OX reveal a (promising) toxicity and efficacy [21,22,36], while the toxicity and efficacy of standard palliative treatment for isolated unresectable colorectal PM seem to be relatively unfavourable [5]. Thereby, repetitive ePIPAC-OX may lead to intraperitoneal disease stabilisation with a low treatment burden and preservation of quality of life of this particular population with an extremely poor life expectancy and a relatively less effective standard palliative treatment.

Enrolment is allowed for patients with an unresected primary tumour and patients who did not receive prior palliative systemic therapy. In these patients, administration of repetitive ePIPAC-OX as a monotherapy could lead to undertreatment and subsequent systemic progression or progression of the primary tumour. However, it is thought that frequent clinical and radiological evaluations detect such progression in a sufficiently early stage. Moreover, patients need to be informed by a medical oncologist about the potential consequences of postponing or discontinuing their standard palliative treatment prior to enrolment. Conclusively, the investigators feel that these controlled circumstances justify enrolment of these patients.

10.5 COMPENSATION FOR INJURY

Both study centres have a liability insurance which is in accordance with article 7 of the WMO. The sponsor (Catharina Hospital, Eindhoven, Netherlands) (also) has an insurance which is in accordance with the legal requirements in the Netherlands (Article 7 WMO). This insurance provides cover for damage to research subjects through injury or death caused by the study. The insurance applies to the damage that becomes apparent during the study or within 4 years after the end of the study.

10.6 INCENTIVES

Except for covering traveling costs, patients do not receive any special incentives, compensation, or treatment through participation.

11. ADMINISTRATIVE ASPECTS, MONITORING AND PUBLICATION

11.1 HANDLING AND STORAGE OF DATA AND DOCUMENTS

All information about potential and enrolled patients is collected, shared, and maintained according to the Dutch law (Wet Bescherming Persoonsgegevens). Outcomes are collected in all patients who receive at least one ePIPAC-OX. All baseline characteristics and clinical outcomes are prospectively collected and entered in an ISO 27001 certified central study database (De Research Manager, Deventer, Netherlands) with study-specific electronic case report forms by a local investigator in each study centre (RJL, ECEW). This ISO 27001 certified system ensures adequate data coding, security, and storage. Questionnaires (quality of life, costs), peritoneal biopsies (histopathological response), and radiological examinations (radiological response) are collected by the coordinating investigator (KPR) throughout the study and centrally analysed after study completion. Human material is stored in the laboratories of the study centres for 15 years.

11.2 MONITORING AND QUALITY ASSURANCE

The study is audited by independent qualified monitors of Clinical Trial Centre Maastricht (Maastricht, Netherlands) as a high risk study according to the brochure 'Kwaliteitsborging mensgebonden onderzoek 2.0' by the Dutch Federation of University Medical Centres. This means that study centres are audited at least three times per year, depending on enrolment, with 100% auditing of the study master file, investigator site files, informed consent forms, eligibility criteria, source data verification, and SAEs/SUSARs.

11.3 AMENDMENTS

A 'substantial amendment' is defined as an amendment to the terms of the METC application, or to the protocol or any other supporting documentation, that is likely to affect to a significant degree:

- the safety or physical or mental integrity of the subjects of the trial;
- the scientific value of the trial;
- the conduct or management of the trial; or
- the quality or safety of any intervention used in the trial.

All substantial amendments are notified to the METC and to the competent authority. Non-substantial amendments are not notified to the accredited METC and the competent authority, but recorded and filed by the sponsor.

11.4 ANNUAL PROGRESS REPORT

The coordinating investigator submits a summary of the progress of the trial to the accredited METC once a year. Information is provided on the date of inclusion of the first subject, numbers of subjects included and numbers of subjects that have completed the trial, serious adverse events/ serious adverse reactions, other problems, and amendments.

11.5 TEMPORARY HALT AND (PREMATURELY) END OF STUDY REPORT

The coordinating investigator notifies the accredited METC and the competent authority of the end of the study within a period of 90 days. The end of the study is defined as the last patient's last visit. The coordinating investigator notifies the METC immediately of a temporary halt of the study, including the reason of such an action. In case the study is ended prematurely, the coordinating investigator notifies the accredited METC and the competent authority within 15 days, including the reasons for the premature termination. Within one year after the end of the study, the coordinating investigator submits a final study report with the results of the study, including any publications/abstracts of the study, to the accredited METC and the Competent Authority.

11.6 PUBLIC DISCLOSURE AND PUBLICATION POLICY

Results of the study are personally communicated to participating patients and communicated to healthcare professionals through publication in peer-reviewed medical journals without any publication restrictions. The main manuscript is written by the coordinating investigator, the principal investigator, the study radiologists, the study pathologists, the study pharmacist (MJD), and a professional English writer, and subsequently revised by all other investigators. Authorship eligibility criteria for manuscript of side studies are not defined *a priori*. The full protocol, Dutch informed consent forms, participant-level datasets, and statistical codes are available upon reasonable request. The study is registered at Clinicaltrials.gov (NCT03246321), ISRCTN (ISRCTN89947480, and NTR (NTR6603).

12. STRUCTURED RISK ANALYSIS

12.1 POTENTIAL ISSUES OF CONCERN

Since the investigational products are registered and used within the indication, chapter 12.1 is skipped.

12.2 SYNTHESIS

The first risk associated with participation is locoregional toxicity of repetitive ePIPAC-OX. However, the locoregional toxicity of PIPAC-OX appears to be low [36]. Furthermore, the dosage of ePIPAC-OX (oxaliplatin 92 mg/m^2 BSA) is much lower than the widely accepted dose of oxaliplatin during HIPEC (300-460 mg/m^2 BSA), which is also used in the COLOPEC trial in both study centres.

The second risk associated with participation is systemic toxicity of repetitive ePIPAC-OX. However, the systemic toxicity of repetitive ePIPAC-OX (92 mg/m^2 BSA every six weeks) is likely to be considerably lower than the systemic toxicity of oxaliplatin during palliative systemic therapy (80-130 mg/m^2 BSA every two to three weeks). When repetitive ePIPAC-OX eventually turns out to be too toxic, this is detected by the clearly defined interim analyses and stopping rules (section 7.6). Finally, patients with contraindications for the planned chemotherapy (e.g. previous allergic reaction) cannot be included in the study.

The last risk is inefficacy of repetitive ePIPAC-OX. However, preliminary results of repetitive ePIPAC-OX reveal a (promising) toxicity and efficacy [21,22,36], while the toxicity and efficacy of standard palliative treatment for isolated unresectable colorectal PM seem to be relatively unfavourable [5]. Thereby, repetitive ePIPAC-OX may lead to intraperitoneal disease stabilisation with a low treatment burden and preservation of quality of life of this particular population with an extremely poor life expectancy and a relatively less effective standard palliative treatment.

Enrolment is allowed for patients with an unresected primary tumour and patients who did not receive prior palliative systemic therapy. In these patients, administration of repetitive ePIPAC-OX as a monotherapy could lead to undertreatment and subsequent systemic progression or progression of

the primary tumour. However, it is thought that frequent clinical and radiological evaluations detect such progression in a sufficiently early stage. Moreover, patients need to be informed by a medical oncologist about the potential consequences of postponing or discontinuing their standard palliative treatment prior to enrolment. Conclusively, the investigators feel that these controlled circumstances justify enrolment of these patients.

13. REFERENCES

[1] van Gestel YR, de Hingh IH, van Herk-Sukel MP, van Erning FN, Beerepoot LV, Wijsman JH, et al. Patterns of metachronous metastases after curative treatment of colorectal cancer. Cancer Epidemiol. 2014;38:448-54.

[2] van der Geest LG, Lam-Boer J, Koopman M, Verhoef C, Elferink MA, de Wilt JH. Nationwide trends in incidence, treatment and survival of colorectal cancer patients with synchronous metastases. Clin Exp Metastasis. 2015;32:457-65.

[3] Razenberg LG, Lemmens VE, Verwaal VJ, Punt CJ, Tanis PJ, Creemers GJ, et al. Challenging the dogma of colorectal peritoneal metastases as an untreatable condition: results of a population-based.

[4] Landelijke werkgroep Gastro Intestinale Tumoren. Richtlijn colorectaal carcinoom. 2014. https://www.oncoline.nl/colorectaalcarcinoom. Accessed 10 Dec 2018.

[5] Franko J, Shi Q, Meyers JP, Maughan TS, Adams RA, Seymour MT, et al. Prognosis of patients with peritoneal metastatic colorectal cancer given systemic therapy: an analysis of individual patient data from prospective randomised trials from the Analysis and Research in Cancers of the Digestive System (ARCAD) database. Lancet Oncol. 2016;17:1709-19.

[6] Sugarbaker PH, Stuart OA, Vidal-Jove J, Pessagno AM, DeBruijn EA. Pharmacokinetics of the peritoneal-plasma barrier after systemic mitomycin C administration. Cancer Treat Res. 1996;82:41-52.

[7] Dedrick RL, Myers CE, Bungay PM, DeVita VT Jr. Pharmacokinetic rationale for peritoneal drug administration in the treatment of ovarian cancer. Cancer Treat Rep. 1978;62:1-11.

[8] Jacquet P, Sugarbaker PH. Peritoneal-plasma-barrier. Cancer Treat Res. 1996;82:53-63.

[9] Dedrick RL, Flessner MF. Pharmacokinetic problems in peritoneal drug administration: tissue penetration and surface exposure. J Natl Cancer Inst. 1997;89:480-7.

[10] Markman M. Limited use of the intraperitoneal route for ovarian cancer – why? Nat Rev Clin Oncol. 2015;12:628-30.

[11] Reymond MA, Solass W. Pressurized IntraPeritoneal Aerosol Chemotherapy – Cancer under Pressure. De Gruyter, 2014.

[12] Reymond MA, Hu B, Garcia A, Reck T, Köckerling F, Hess J, et al. Feasibility of therapeutic pneumoperitoneum in a large animal model using a microvaporisator. Surg Endosc. 2000;14:51-5.

[13] Jacquet P, Stuart OA, Chang D, Sugarbaker PH. Effects of intra-abdominal pressure on pharmacokinetics and tissue distribution of doxorubicin after intraperitoneal administration.

[14] Esquis P, Consolo D, Magnin G, Pointaire P, Ynsa MD, Beltramo JL, et al. High intra-abdominal pressure enhances the penetration and antitumor effect of intraperitoneal cisplatin on experimental peritoneal carcinomatosis. Ann Surg. 2006;244:106-112.

[15] Solass W, Herbette A, Schwarz T, Hetzel A, Sun JS, Dutreix M, et al. Therapeutic approach of human peritoneal carcinomatosis with Dbait in combination with capnoperitoneum: proof of concept. Surg Endosc. 2012;26:847-52.

[16] Solass W, Hetzel A, Nadiradze G, Sagynaliev E, Reymond MA. Description of a novel approach for intraperitoneal drug delivery and the related device. Surg Endosc. 2012;26:1849-55.

[17] Facy O, Al Samman S, Magnin G, Ghiringhelli F, Ladoire S, Chauffert B, et al. High pressure enhances the effect of hyperthermia in intraperitoneal chemotherapy with oxaliplatin: an experimental study. Ann Surg. 2012;256:1084-8.

[18] Solass W, Kerb R, Mürdter T, Giger-Pabst U, Strumberg D, Tempfer C, et al. Intraperitoneal chemotehrapy of peritoneal carcinomatosis using pressurized aerosol as an alternative to liquid solution: first evidence for efficacy. Ann Surg Oncol. 2014;21:553-9.

[19] Blanco A, Giger-Pabst U, Solass W, Zieren J, Reymond MA. Renal and hepatic toxicities after pressurized intraperitioneal aerosol chemotherapy (PIPAC). Ann Surg Oncol. 2013;20:2311-6.

[20] Eveno C, Haidara A, Ali I, Pimpie C, Mirshahi M, Pocard M. Experimental pharmacokinetics evaluation of chemotherapy delivery by PIPAC for colon cancer: first evidence for efficacy. Pleura and Peritoneum. 2017;2:103-110.

[21] Grass F, Vuagnieaux A, Teixeira-Farinha H, Lehmann K, Demartines N, Hübner M, et al. Systematic review of pressurized intraperitoneal aerosol chemotherapy for the treatment of advanced peritoneal carcinomatosis. Br J Surg. 2017;104:669-78.

[22] Tempfer C, Giger-Pabst U, Hilal Z, Dogan A, Rezniczek GA. Pressurized intraperitoneal aerosol chemotherapy (PIPAC) for peritoneal carcinomatosis: systematic review of clinical and experimental evidence with special emphasis on ovarian cancer. Arch Gynecol Obstet. 2018;243-57.

[23] Nowacki M, Alyami M, Villeneuve L, Mercier F, Hübner M, Willaert W, et al. Multicenter comprehensive methodological and technical analysis of 832 pressurized intraperitoneal aerosol chemotherapy (PIPAC) interventions performed in 349 patients for peritoneal carcinomatosis treatment: an international survey study. Eur J Surg Oncol. 2018;33:991-6.

[24] Willaert W, Sessink P, Ceelen W. Occupational safety of pressurized intraperitoneal aerosol chemotherapy (PIPAC). Pleura and Peritoneum. 2017;2:121-8.

[25] Graversen M, Lundell L, Fristrup C, Pfeiffer P, Mortensen MB. Pressurized intraperitoneal aerosol chemotherapy (PIPAC) as an outpatient procedure. Pleura and Peritoneum. 2018;20180128.

[26] Kakchekeeva T, Demtröder C, Herath NI, Griffiths D, Torkington J, Solass W, et al. In vivo feasibility of electrostatic precipitation as an adjunct to pressurized intraperitoneal aerosol chemotherapy (ePIPAC). Ann Surg Oncol. 2016;23:592-8.

[27] Odendahl K, Solass W, Demtröder C, Giger-Pabst U, Zieren J, Tempfer C, et al. Quality of life of patients with end-stage peritoneal metastasis treated with pressurized intraperitoneal aerosol chemotherapy (PIPAC). Eur J Surg Oncol. 2015;41:1379-85.

[28] Robella M, Vaira M, de Simone M. Safety and feasibility of pressurized intraperitoneal aerosol chemotherapy (PIPAC) associated with systemic chemotherapy: an innovative approach to treat peritoneal carcinomatosis. World J Surg Oncol. 2016;14:128.

[29] Teixeira Farinha H, Grass F, Kefleyesus A, Achtari C, Romain B, Montemurro M, et al. Impact of pressurized intraperitoneal aerosol chemotherapy on quality of life and symptoms in patients with peritoneal carcinomatosis: a retrospective cohort study. Gastroenterol Res Pract. 2017;2017:4596176.

[30] Hübner M, Teixeira Farinha H, Grass F, Wolfer A, Mathevet P, Hahnloser D, et al. Feasibility and safety or pressurized intreapeirtoneal aerosol chemotherapy for peritoneal carcinomatosis: a retrospective cohort study. Gastroenterol Res Pract. 217;2017:6852749.

[31] Hübner M, Grass F, Teixeira-Farinha H, Pache B, Mathevet P, Demartines N. Pressurized intraperitoneal aerosol chemotherapy – practical aspects. Eur J Surg Oncol. 2017;43:1102-9.

[32] Alyami M, Gagniere J, Sgarbura O, Cabelguenne D, Villeneuve L, Pezet D, et al. Multicentric initial experience with the use of the pressurized intraperitoneal aerosol chemotherapy (PIPAC) in the management of unresectable peritoneal carcinomatosis. Eur J Surg Oncol. 2017;43:2178-83.

[33] Graversen M, Detlefsen S, Bjerregaard JK, Fristrup CW, Pfeiffer P, Mortensen MB. Prospective, single-center implementation and response evaluation of pressurized intraperitoneal aerosol chemotherapy (PIPAC) for peritoneal metastasis. Ther Adv Med Oncol. 2018;10:1758835918777036.

[34] Kurtz F, Struller F, Horvath P, Solass W, Bösmüller H, Königsrainer A. Feasibility, safety, and efficacy of pressurized intraperitoneal aerosol chemotherapy (PIPAC) for peritoneal metastasis: a registry study. Gastroenterol Res Pract. 2018;2018:2743985.

[35] Teixeira Farinha H, Grass F, Labgaa I, Pache B, Demartines N, Hübner M. Inflammatory response and toxicity after pressurized intraperitoneal aerosol chemotherapy. J Cancer. 2018;9:13-20.

[36] Demtröder C, Solass W, Zieren J, Strumberg D, Giger-Pabst U, Reymond MA. Pressurized intraperitoneal aerosol chemotherapy with oxaliplatin in colorectal peritoneal metastasis. Colorectal Dis. 2016;18:364-71.

[37] Graversen M, Detlefsen S, Pfeiffer P, Lundell L, Mortsensen MB. Severe peritoneal sclerosis after repeated pressurized intraperitoneal aerosol chemotherapy with oxaliplatin (PIPAC OX): report of two cases and literature survey. Clin Exp Metastasis. 2018;35:103-8.

[38] Siebert M, Alyami M, Mercier F, Gallice C, Villeneuve L, Bérard F, et al. Severe hypersensitivity reactions to platinum compounds post-pressurized intraperitoneal aerosol chemotherapy (PIPAC): first literature report. Cancer Chemother Pharmacol. 2018. doi: 10.1007/s00280-018-3740-3.

[39] Clinicaltrials.gov. www.clinicaltrials.gov. Accessed 10 Dec 2018.

[40] Solass W, Giger-Pabst U, Zieren J, Reymond MA. Pressurized intraperitoneal aerosol chemotherapy (PIPAC): occupational health and safety aspects. Ann Surg Oncol. 2013;20:3504-11.

[41] Graversen M, Pedersen PB, Mortensen MB. Environmental safety during the administration of Pressurized intraperitoneal aerosol chemotherapy (PIPAC). Pleura and Peritoneum;2016:203-8.

[42] Ndaw S, Hanser O, Kenepekian V, Vidal M, Melczer M, Remy A, et al. Occupational exposure to platinum drugs during intraperitoneal chemotherapy. Biomonitoring and surface contamination. Toxicol Lett. 2018;298:171-6.

[43] Ametsbichler P, Böhlandt A, Nowak D, Schierl R. Occupational exposure to cisplatin/oxaliplatin during pressurized intraperitoneal aerosol chemotherapy (PIPAC)? Eur J Surg Oncol. 2018;44:1793-9.

[44] Elias D, Bonnay M, Puizillou JM, Antoun S, Demirdjian S, El OA, et al. Heated intra-operative intraperitoneal oxaliplatin after complete resection of peritoneal carcinomatosis: pharmacokinetics and tissue distribution. Ann Oncol. 2002;13:267-72.

[45] Giachetti S, Perpoint B, Zidani R, le Bail N, Faggiuolo R, Focan C, et al. Phase III multicentre randomized trial of oxaliplatin added to chronomodulated fluorouracil-leucovorin as first-line treatment of metastatic colorectal cancer. J Clin Oncol. 2000;18:136-47.

[46] Lemmens VE, Klaver YL, Verwaal VJ, Rutten HJ, Coerbergh JW, de Hingh IH. Predictors and survival of synchronous peritoneal carcinomatosis of colorectal origin: a population-based study. Int J Cancer. 2011;128:2717-25.

[47] van Gestel YR, Thomassen I, Lemmens VE, Pruijt JF, van Herk-Sukel MP, Rutten HJ, et al. Metachronous peritoneal carcinomatosis after curative treatment of colorectal cancer. Eur J Surg Oncol. 2014;40:963-9.

[48] Giger-Pabst U, Tempfer CB. How to perform safe and technically optimized pressurized intraperitoneal aerosol chemotherapy (PIPAC): experience after a consecutive series of 1200 procedures. J Gastrointest Surg. 2018;22:2187-93.

[49] Zühlke HV, Lorenz EM, Straub EM, Savvas V. Pathophysiology and classification of adhesions. Langenbecks Arch Chir II Verh Dtsch Ges Chir. 1990:1009-16.

[50] Jacquet P, Sugarbaker PH. Clinical research methodologies in diagnosis and staging of patients with peritoneal carcinomatosis. Cancer Treat Res. 1996;82:359-74.

[51] Herdman M, Gudex C, Lloyd A, Janssen M, Kind P, Parkin D, et al. Development and preliminary testing of the new five-level version of EQ-5D (EQ-5D-5L). Qual Life Res. 2011;20:1727-36.

[52] Aaronson NK, Ahmedzai S, Bergman B, Bullinger M, Cull A, Duez NJ, et al. The European Organisation for Research and Treatment of Cancer QLQ-C30: a quality-of-life instrument for use in international clinical trials in oncology. J Natl Cancer Inst. 1993;85:365-76.

[53] Stiggelbout AM, Kunneman M, Baas-Thijssen MC, Neijenhuis PA, Loor AK, Jägers S, et al. The EORTC QLQ-CR29 quality of life questionnaire for colorectal cancer: validation of the Dutch version. Qual Life Res. 2016;25:1853-8.

[54] Bouwmans C, Krol M, Severens H, Koopmanschap M, Brouwer W, Hakkaart-van Roijen L. The iMTA Productivity Cost Questionnaire: a standardized instrument for measuring and valuing health-related productivity losses. Value Health. 2015;18:753-8.

[55] iMTA: questionnaires. https://www.imta.nl/questionnaires/. Accessed 10 Dec 2018.

[56] Elias D, Bonnay M, Puizillou JM, et al. Heated intra-operative intraperitoneal oxaliplatin after complete resection of peritoneal carcinomatosis: pharmacokinetics and tissue distribution. Ann Oncol. 2002;13(2):267-272.

[57] Elias D, el Otmany A, Bonnay M, et al. Human pharmacokinetic study of heated intraperitoneal oxaliplatin in increasingly hypotonic solutions after complete resection of peritoneal carcinomatosis. Oncology. 2002;63(4):346-352.

[58] Elias D, Sideris L. Pharmacokinetics of heated intraoperative intraperitoneal oxaliplatin after complete resection of peritoneal carcinomatosis. Surg Oncol Clin N Am.2003;12(3):755-769.

[59] Elias D, Pocard M, Sideris L, et al. Preliminary results of intraperitoneal chemohyperthermia with oxaliplatin in peritoneal carcinomatosis of colorectal origin. Br J Surg. 2004;91(4):455-456.

[60] Elias D, Sideris L, Pocard M, et al. Efficacy of intraperitoneal chemohyperthermia with oxaliplatin in colorectal peritoneal carcinomatosis. Preliminary results in 24 patients. Ann Oncol. 2004;15(5):781-785.

[61] Elias D, Matsuhisa T, Sideris L, et al. Heated intra-operative intraperitoneal oxaliplatin plus irinotecan after complete resection of peritoneal carcinomatosis: pharmacokinetics, tissue distribution and tolerance. Ann Oncol. 2004;15(10):1558-1565.

[62] Elias D, Raynard B, Boige V, et al. Impact of the extent and duration of cytoreductive surgery on postoperative hematological toxicity after intraperitoneal chemohyperthermia for peritoneal carcinomatosis. J Surg Oncol. 2005;90(4):220-225.

[63] Mura G, Framarini M, Milandri C, et al. Intraperitoneal chemotherapy with oxaliplatin after complete cytoreduction for peritoneal carcinomatosis from colorectal carcinoma: preliminary experience. Suppl Tumor. 2005;4(3):S111-2.

[64] Elias D, Raynard B, Bonnay M, et al. Heated intra-operative intraperitoneal oxaliplatin alone and in combination with intraperitoneal irinotecan: pharmacologic studies. Eur J Surg Oncol. 2006;32(6):607-613.

[65] Rouers A, Laurent S, Detroz B, et al. Cytoreductive surgery and hyperthermic intraperitoneal chemotherapy for colorectal peritoneal carcinomatosis: higher complication rate for oxaliplatin compared to mitomycin C. Acta Chir Belg. 2006;106(3):302-306.

[66] Elias D, Raynard B, Farkhondeh F, et al. Peritoneal carcinomatosis of colorectal origin. Gastroenterol Clin Biol. 2006;30(10):1200-1204.

[67] Elias D, Benizri E, di Pietrantonio D, et al. Comparison of two kinds of intraperitoneal chemotherapy following complete cytoreductive surgery of colorectal peritoneal carcinomatosis. Ann Surg Oncol. 2007;14(2):509-514.

[68] Cavaliere F, Valle M, de Simone M, et al. 120 peritoneal carcinomatoses from colorectal cancer treated with peritonectomy and intra-abdominal chemohyperthermia: a S.I.T.I.L.O. multicentric study. In Vivo. 2006;20(6A):747-750.

[69] Elias D, Goéré D, Blot F, et al. Optimization of hyperthermic intraperitoneal chemotherapy with oxaliplatin plus irinotecan at 43 degrees C after complete cytoreductive surgery: mortality and morbidity in 106 consecutive patients. Ann Surg Oncol. 2007;14(6):1818-1824.

[70] Mura G, Framarini M, Vagliasindi A, et al. Preliminary experience with hyperthermic intraperitoneal chemo-perfusion with oxaliplatin for the treatment of peritoneal carcinomatosis due to colorectal carcinoma. Chir Ital. 2007;59(2):217-223.

[71] Ceelen WP, Peeters M, Houtmeyers P, et al. Safety and efficacy of hyperthermic intraperitoneal chemoperfusion with high-dose oxaliplatin in patients with peritoneal carcinomatosis. Ann Surg Oncol. 2008;15(2):535-541.

[72] Ferron G, Dattez S, Gladieff L, et al. Pharmacokinetics of heated intraperitoneal oxaliplatin. Cancer Chemother Pharmacol. 2008;62(4):679-683.

[73] Elias D, Bedard V, Bouzid T, et al. Malignant peritoneal mesothelioma: treatment with maximal cytoreductive surgery plus intraperitoneal chemotherapy. Gastroenterol Clin Biol. 2007;31(10):784-788.

[74] Stewart JH 4th, Shen P, Russell G, et al. A phase I trial of oxaliplatin for intraperitoneal hyperthermic chemoperfusion for the treatment of peritoneal surface dissemination from colorectal and appendiceal cancers. Ann Surg Oncol. 2008;15(8):2137-2145.

[75] Mahteme H, Wallin I, Glimelius B, et al. Systemic exposure of the parent drug oxaliplatin during hyperthermic intraperitoneal perfusion. Eur J Clin Pharmacol. 2008;64(9):907-911.

[76] Marcotte E, Sideris L, Drolet P, et al. Hyperthermic intraperitoneal chemotherapy with oxaliplatin for peritoneal carcinomatosis arising from appendix: preliminary results of a survival analysis. Ann Surg Oncol. 2008;15(10):2701-2708.

[77] Elias D, Honoré C, Ciuchendéa R, et al. Peritoneal pseudomyxoma: results of a systematic policy of complete cytoreductive surgery and hyperthermic intraperitoneal chemotherapy. Br J Surg. 2008;95(9):1164-1171.

[78] Elias D, Lefevre JH, Chevalier J, et al. Complete cytoreductive surgery plus intraperitoneal chemohyperthermia with oxaliplatin for peritoneal carcinomatosis of colorectal origin. J Clin Oncol. 2009;27(5):681-685.

[79] Fagotti A, Paris I, Grimolizzi F, et al. Secondary cytoreduction plus oxaliplatin-based HIPEC in platinum-sensitive recurrent ovarian cancer patients: a pilot study. Gynecol Oncol. 2009;113(3):335-340.

[80] Sideris L, Mitchell A, Drolet P, et al. Surgical cytoreduction and intraperitoneal chemotherapy for peritoneal carcinomatosis arising from the appendix. Can J Surg. 2009;52(2):135-141.

[81] Ceelen WP, van Nieuwenhove Y, van Belle S, et al. Cytoreduction and hyperthermic intraperitoneal chemoperfusion in women with heavily pretreated recurrent ovarian cancer. Ann Surg Oncol. 2012;19(7):2352-2359.

[82] Carrabin N, Mithieux F, Meeus P, et al. Hyperthermic intraperitoneal chemotherapy with oxaliplatin and without adjuvant chemotherapy in stage IIIC ovarian cancer. Bull Cancer. 2010;97(4):E23-32.

[83] Ba MC, Cui SZ, Lin SQ, et al. Chemotherapy with laparoscope-assisted continuous circulatory hyperthermic intraperitoneal perfusion for malignant ascites. World J Gastroenterol. 2010;16(15):1901-1907.

[84] Bretcha-Boix P, Farré-Alegre J, Sureda M, et al. Cytoreductive surgery and perioperative intraperitoneal chemotherapy in patients with peritoneal carcinomatosis of colonic origin: outcomes after 7 years' experience of a new centre for peritoneal surface malignancies. Clin Transl Oncol. 2010;12(6):437-442.

[85] Rueth NM, Murray SE, Huddleston SJ, et al. Severe electrolyte disturbances after hyperthermic intraperitoneal chemotherapy: oxaliplatin versus mitomycin C. Ann Surg Oncol. 2012;18(1):174-180.

[86] Frenel S, Leux C, Pouplin L, et al. Oxaliplatin-based hyperthermic intraperitoneal chemotherapy in primary or recurrent epithelial ovarian cancer: a pilot study of 31 patients. J Surg Oncol. 2011;103(1):10-6.

[87] Fagotti A, Costantini B, Vizzielli G, et al. HIPEC in recurrent ovarian cancer patients: morbidity-related treatment and long-term analysis of clinical outcome. Gynecol Oncol. 2011;122(2):221-225.

[88] Quenet F, Goéré D, Mehta SS, et al. Results of two bi-institutional prospective studies using intraperitoneal oxaliplatin with or without irinotecan during HIPEC after cytoreductive surgery for colorectal carcinomatosis. Ann Surg. 2011;254(2):294-301.

[89] Hompes D, d'Hoore A, van Cutsem E, et al. The treatment of peritoneal carcinomatosis of colorectal cancer with complete cytoreductive surgery and hyperthermic intraperitoneal peroperative chemotherapy (HIPEC) with oxaliplatin: a Belgian multicenter prospective phase II clinical study. Ann Surg Oncol. 2012;19(7):2186-2194.

[90] Votanopoulos K, Ihemelandu C, Shen P, et al. A comparison of hematologic toxicity profiles after heated intraperitoneal chemotherapy with oxaliplatin and mitomycin C. J Surg Res. 2013;179(1):e133-9.

[91] Glockzin G, von Breitenbuch P, Schlitt HJ, et al. Treatment-related morbidity and toxicity of CRS and oxaliplatin-based HIPEC compared to a mitomycin and doxorubicin-based HIPEC protocol in patients with peritoneal carcinomatosis: a matched-pair analysis. J Surg Oncol. 2013;107(6):574-578.

[92] Ceelen W, de Somer F, van Nieuwenhove Y, et al. Effect of perfusion temperature on glucose and electrolyte transport during hyperthermic intraperitoneal chemoperfusion (HIPEC) with oxaliplatin. Eur J Surg Oncol. 2013;39(7):754-759.

[93] Turrini O, Lambaudie E, Faucher M, et al. Initial experience with hyperthermic intraperitoneal chemotherapy. Arch Surg. 2012;147(10):919-923.

[94] McConnell YJ, Mack LA, Francis WP, et al. HIPEC + EPIC versus HIPEC alone: differences in major complications following cytoreduction surgery for peritoneal malignancy. J Surg Oncol. 2013;107(6):591-596.

[95] Pérez-Ruixo C, Valenzuela B, Peris JE, et al. Population pharmacokinetics of hyperthermic intraperitoneal oxaliplatin in patients with peritoneal carcinomatosis after cytoreductive surgery. Cancer Chemother Pharmacol. 2013;71(3):693-704.

[96] Pérez-Ruixo C, Valenzuela B, Peris JE, et al. Neutrophil dynamics in peritoneal carcinomatosis patients treated with cytoreductive surgery and hyperthermic intraperitoneal oxaliplatin. Clin Pharmacokinet. 2013;52(12):1111-25.

[97] Schwarz L, Bridoux V, Veber V, et al. Hematophagocytic syndrome: an unusual and underestimated complication of cytoreduction surgery with heated intraperitoneal oxaliplatin. Ann Surg Oncol. 2013;20(12):3919-26.

[98] Gouy S, Uzan C, Pautier P, et al. Results of oxaliplatin-based hyperthermic intraperitoneal chemotherapy in recurrent ovarian granulosa cell tumors. Eur J Obstet Gynecol Reprod Biol. 2013;170(2):464-7.

[99] Gervais MK, Dubé P, McConnell Y, et al. Cytoreductive surgery plus hyperthermic intraperitoneal chemotherapy with oxaliplatin for peritoneal carcinomatosis arising from colorectal cancer. J Surg Oncol. 2013;108(7):438-443.

[100] Hompes D, D'Hoore A, Wolthuis A, et al. The use of oxaliplatin of mitomycin C in HIPEC treatment for peritoneal carcinomatosis from colorectal cancer: a comparative study. J Surg Oncol. 2014;109(6):527-532.

[101] Fagotti A, Petrillo M, Costantini B, et al. Minimally invasive secondary cytoreduction plus HIPEC for recurrent ovarian cancer: a case series. Gynecol Oncol. 2014;132(2):303-306.

[102] Shimizu T, Sonoda H, Murata S, et al. Hyperthermic intraperitoneal chemotherapy using a combination of mitomycin C, 5-fluorouracil, and oxaliplatin in patients at high risk of colorectal peritoneal metastasis: a phase I clinical study. Eur J Surg Oncol. 2014;40(5):521-528.

[103] Nikolic S, Dzodic R, Zegarac M, et al. Survival prognostic factors in patients with colorectal peritoneal carcinomatosis traeted with cytoreductive surgery and intraoperative hyperthermic intraperitoneal chemotherapy: a single institution experience. J BUON. 2014;19(1):66-74.

[104] Pérez-Ruixo C, Peris JE, Escudero-Ortiz V, et al. Rate and extent of oxaliplatin absorption after hyperthermic intraperitoneal administration in peritoneal carcinomatosis patients. Cancer Chemother Pharmacol. 2014;73(5):1009-1020.

[105] Muller H, Hotopp T, Tofeili A, et al. Systemic chemotherapy using FLOT-regimen combined with cytoreductive surgery plus HIPEC for treatment of peritoneal metastasized gastric cancer. Hepatogastroenterology. 2014;61(131):703-706.

[106] Chalret du Rieu Q, White-Koning M, Picaud L, et al. Population pharmacokinetics of peritoneal, plasma ultrafiltrated and protein-bound oxaliplatin concentrations in patients with disseminated peritoneal cancer after intraperitoneal hyperthermic chemoperfusion of oxaliplatin following cytoreductive surgery: correlation between oxaliplatin exposure and thrombocytopenia. Cancer Chemother Pharmacol. 2014;74(3):571-582.

[107] Prada-Villaverde A, Esquivel J, Lowy AM, et al. The American Society of Peritoneal Surface Malignancies evaluation of HIPEC with mitomycin C versus oxaliplatin in 539 patients with colon cancer undergoing a complete cytoreductive surgery. J Surg Oncol. 2014;110(7):779-785.

[108] Glockzin G, Gerken M, Lang SA, et al. Oxaliplatin-based versus irinotecan-based hyperthermic intraperitoneal chemotherapy (HIPEC) in patients with peritoneal metastases from appendiceal and colorectal cancer: a retrospective analysis. BMC Cancer. 2014;14:807.

[109] Marcotte E, Dubé P, Drolet P, et al. Hyperthermic intraperitoneal chemotherapy with oxaliplatin as treatment for peritoneal carcinomatosis arising from the appendix and pseudomyxoma peritonei: a survival analysis.

[110] Hubert J, Thiboutot E, Dubé P, et al. Cytoreductive surgery and hyperthermic intraperitoneal chemotherapy with oxaliplatin for peritoneal mesothelioma: preliminary resutls and survival analysis. Surg Oncol. 2015;24(1):41-46.

[111] Lam JY, McConnell YJ, Rivard JD, et al. Hyperthermic intraperitoneal chemotherapy + early postoperative intraperitoneal chemotherapy versus hyperthermic intraperitoneal chemotherapy alone: assessment of survival outcomes for colorectal and high-grade appendiceal peritoneal carcinomatosis. Am J Surg. 2015;210(3):424-430.

[112] Petrillo M, de Iaco P, Cianci S, et al. Long-term survival for platinum-sensitive recurrent ovarian cancer patients treated with secondary cytoreductive surgery plus hyperthermic intraperitoneal chemotherapy (HIPEC). Ann Surg Oncol. 2016;23(5);1660-1665.

[113] Charrier T, Passot G, Peron J, et al. Cytoreductive surgery combined with hyperthermic intraperitoneal chemotherapy with oxaliplatin increases the risk of postoperative hemorrhagic complications: analysis of predictive factors. Ann Surg Oncol. 2016;23(7):2315-2322.

[114] Horvath P, Beckert S, Struller F, et al. Incidence of leukopenia after intraperitoneal vs. combined intravenous/intraperitoneal chemotherapy in pseudomyxoma peritonei. World J Gastrointest Pharmacol Ther. 2016;7(3):434-439.

[115] Leung V, Huo YR, Liauw W, et al. Oxaliplatin versus mitomycin C for HIPEC in colorectal cancer peritoneal carcinomatosis. Eur J Surg Oncol. 2017;43(1):144-149.

[116] Löffler MW, Schuster H, Zeck A, et al. Pharmacodynamics of oxaliplatin-derived platinum compounds during hyperthermic intraperitoneal chemotherapy (HIPEC): an emerging aspect supporting the rational design of treatment protocols. Ann Surg Oncol. 2017;24:1650-7.

[117] Delhorme JB, Sattler L, Severac F, et al. Prognostic factors of hemorrhagic complications after oxaliplatin-based hyperthermic intraperitoneal chemotherapy: toward routine preoperative dosage of von willebrand factor? Eur J Surg Oncol. 2017;43:1095-101.

[118] Willaert W, van der Speeten K, Liberale G, et al. BEV-IP: Perioperative chemotherapy with bevacizumab in patients undergoing cytoreduction and intraperitoneal chemoperfusion for colorectal carcinomatosis. BMC Cancer. 2015;15:980.

[119] Klaver CE, Musters GD, Bemelman WA, et al. Adjuvant hyperthermic intraperitoneal chemotherapy (HIPEC) in patients with colon cancer at high risk of peritoneal carcinomatosis; the COLOPEC randomized multicenter trial. BMC Cancer. 2015;15:428.

[120] Common Terminology Criteria for Adverse Events (CTCAE) v4.0. National Cancer Institute. 2009. https://evs.nci.nih.gov/ftp1/CTCAE/CTCAE_4.03/Archive/CTCAE_4.0_2009-05-29_QuickReference_8.5x11.pdf. Accessed 10 Dec 2018.

[121] Dindo D, Demartines N, Clavien PA. Classification of surgical complications: a new proposal with evaluation in a cohort of 6336 patients and results of a survey. Ann Surg. 2004;240:205-13.

[122] Solass W, Sempoux C, Detlefsen S, Carr NJ, Bibeau F. Peritoneal sampling and histological assessment of therapeutic response in peritoneal metastasis: proposal of the Peritoneal Regression Grading Score (PRGS). Pleura and Peritoneum. 2016;

14. ATTACHMENTS

14.1 FIRST CHECKLIST

1. Dik perifeer infuus op OK? ☐
2. Intraveneuze chemotherapie (5-FU, leucovorine, 3x NaCl) inclusief perfusoren op OK? ☐
3. Heparinebuizen, EDTA-buizen, kinetieklijst, en PA-formulieren op OK? ☐
4. Ultravision® generator op OK? ☐
5. Nemoto Rempress® en afstandsbediening op OK, en ingesteld op PIPAC-programma? ☐
6. Intraperitoneale chemotherapie (oxaliplatine) op OK, geplaatst in Nemoto Rempress®? ☐
7. Blauwe onderleggers en Septoboxen aanwezig? ☐
8. Gekoppelde infuuslijnen met 60 cc NaCl spuit op OK? ☐
9. Drie functionerende schermen buiten OK? ☐
10. Kar met HIPEC-kleding en cytostaticabordjes buiten OK? ☐
11. Extra anesthesiekar buiten OK? ☐
12. Tafel OK-assistent compleet? Met in ieder geval: ☐
    a. 1x HIPEC-kleding? ☐
    b. 2x ballontrocar met kleine ballon? ☐
    c. Zuigslang voor opzuigen ascites? ☐
    d. NaCl injectie (bij geen ascites)? ☐
    e. Potjes voor opvangen PA (4x) en ascites (2x)? ☐
    f. Biopsietang en cliptang? ☐
    g. Ultravision® Ionwand™? ☐
    h. Dopjes voor op trocars? ☐
    i. CapnoPen® met hogedruklijn? ☐
    j. Extra camerahoes? ☐
    k. Materiaal om CapnoPen® en camera te fixeren? ☐
    l. Filstersysteem? ☐

14.2 SECOND CHECKLIST

1. Zühlke score bepaald? ☐
2. Ascitesvolume gedocumenteerd? ☐
3. Ascites opgevangen, deel ingestuurd voor translationeel + cytologie? ☐
4. Biopt peritoneale metastasen ingestuurd voor translationeel + histologie? ☐
5. Bioptlocaties gemarkeerd met clip? ☐
6. PCI per regio bepaald en foto's van beoordeelde regio's gemaakt? ☐
7. Ionwand™ geplaatst in vrije positie (>2cm), verbonden met generator (uit) ☐
8. Beide trocars afgesloten met dopjes? ☐
9. CO_2 insoufflatie op CapnoPen®-trocar en AAN? ☐
10. Filtersysteem op camera-trocar en CO_2 exsoufflatie UIT? ☐
11. Filtersysteem goed aangesloten? ☐
12. Camera gefixeerd in abdomen, met tip van CapnoPen® in beeld? ☐
13. CapnoPen® gefixeerd in abdomen, met tip in vrije positie? ☐

14. Capnoperitoneum lekvrij (flow <0.2 L/min) op 12 mmHg? ☐
15. Hogedruklijn van CapnoPen® verbonden met spuit, connectie beschermd met camerahoes? ☐
16. Blauwe onderleggers onder OK-tafel en spuit gepositioneerd boven septobox? ☐
17. Nemoto Rempress® geïnstalleerd op PIPAC-programma (150 ml, 200 psi, 0.5 ml/sec)? ☐
18. Extra pijnstilling en spierverslapping gegeven door anesthesie? ☐
19. Gekoppelde infuuslijnen met 60 cc NaCl naar buiten OK? ☐
20. Infuuszakken en damp voldoende gevuld? ☐
21. Scherm Nemoto Rempress® gedraaid naar raam? ☐
22. 5-FU / leucovorine helemaal ingelopen? ☐
23. Instrumenten voor sluiten klaargelegd? ☐
24. Cytostatica-bordjes op de deur? ☐
25. Eén iemand in beschermende kleding? ☐

14.3 PROTOCOL FOR (NURSE) ANAESTHESIOLOGISTS

Doelstelling

Het geven van anesthesiologische zorg aan de patiënt, met inachtneming van alle veiligheidsmaatregelen bij het peroperatief gebruik van cytostatica.

Uitvoerenden

Anesthesiologen,
Arts-assistenten anesthesiologie
Anesthesie medewerkers

Algemene opmerkingen

Een PIPAC-operatie is een laparoscopische palliatieve ingreep voor patiënten met uitgebreide peritoneale metastasen.
Ingreep wordt gedaan op OK 4
Medewerkers die zwanger zijn of borstvoeding geven worden niet ingezet bij deze operatie.
Handhygiëne dient plaats te vinden na elke handeling tijdens de gehele operatieprocedure en bij wisseling van patiënt.
Alle scopen (laryngoscoopbladen, flexibele bronchoscopen, bonfils) moeten gereinigd worden volgens het protocol scopen en intubatiemateriaal.

Benodigdheden

Tube
Perifeer infuus met groot lumen i.v.m. bloedafname
BIS
Temperatuurprobe
Warmtedeken tube
Disposable RR band
NMT monitoring
Extra perfusor

Controle van de werking van de 3 beeldschermen in de regiekamer naast OK 4 (Monitor, camera en laparoscopiebeeld)
Dienstkar van de anesthesie op de gang voor de deur
4-5 perfusorlijnen aan elkaar gekoppeld met op het uiteinde een 60ml spuit met NaCl

Werkwijze

Inleiding:
Rugligging, armen uit.
Tafel horizontaal

Inleiden met:
Propofol
Rocuronium 0,5 mg/kg
Sufentanil
Ketanest 0.25 mg/kg
Dexamethason 8 mg
Granisetron 1 mg
Intubatie
Normothermie nastreven
Onderhoud van de anesthesie m.b.v. damp

Operatie procedure:
De operatie bestaat uit 3 fasen:
- 1e fase: Inbrengen trocars en laparoscopie, bepalen van PCI, nemen van biopten, cytologie, en plaatsen van de vernevelaar (CapnoPen).

Indien na het inbrengen van trocars en camera blijkt dat de PIPAC daadwerkelijk door gaat krijgt de patiënt direct IV cytostatica. Hierbij worden d.m.v. een perfusor voor gevulde spuiten met cytostatica toegediend:
Spuit met folinezuur in laten lopen in 10 minuten
Het systeem door spoelen met meegeleverde spuit met NaCl.
Hierna de spuit met fluorouracil in laten lopen in 15 minuten.
Het systeem door spoelen met de andere meegeleverde spuit met NaCl.
Spuiten en toedieningslijn los koppelen van het infuus en in de septobox (blauwe bak) weg gooien. Belangrijk: werk met handschoenen aan en een beschermbril!
Patiënt opladen met 0,1mg/Kg Morfine bij het vernevelen met Oxaliplatine

- 2e fase: Vernevelen van het cytostaticum

Neemt ongeveer 30 minuten in beslag. Gedurende deze tijd is de patiënt alleen op de kamer
Voor start vernevelen 10mg rocuronium en zn. extra pijnstilling toedienen.
Controle van gevuld zijn van de verdamper en infuuszakken.
Aan het infuus de verlengde perfusorlijnen met de 60ml spuit maken en deze tussen de deur of via het luikje buiten de OK leiden.
Vlak voor het starten van het vernevelen verlaat iedereen de kamer.
Observatie van alles via de beeldschermen op de regiekamer en het raam naar OK 4.

Medicatie toedienen via de verlengde perfusorlijn indien nodig en doorspuiten met de 60ml spuit met NaCl.
In geval van een calamiteit waarbij de kamer betreden moet worden gaat de operatie assistent/chirurg eerst naar binnen met beschermende kleding aan, stopt de CO2 en zuigt de gassen uit de buik. De kamer is dan met beschermende kleding aan te betreden.

- 3^{e} fase: Verwijderen van de vernevelaar, trocars en sluiten van de wonden

Patiënten worden op de kamer geextubeerd
Eventueel spierverslapping antagoneren. (NMT monitoring)

Postoperatief:
Excreten als besmet beschouwen
Pijnstilling in principe schema 1.1, en schema 2.1 indien oxaliplatine gebruikt is.

Hieronder vermelde maatregelen i.v.m. besmettingsgevaar met cytostatica blijven van kracht tot uit toxiciteitsmetingen blijkt dat het niet nodig is.

Persoonlijke bescherming:
Tijdens en na afloop van het vernevelen persoonlijke beschermingsmiddelen gebruiken:
Spatbril, handschoenen (onsteriel), overschort (onsteriel), oversloffen en FFP3 masker

Specifieke patiëntenzorg:
Eventuele samples mogen in het sample apparaat op de OK, spuit moet wel mee terug naar OK genomen worden en daar in septobox (blauwe bak).
Tijdens en na het vernevelen / chemo IV toediening scheidt de patiënt gedurende 2 tot 7 dagen cytostatica uit via excreten: urine, faeces, slijm, braaksel, bloed, drainvocht en zweet. (Afhankelijk van gebruikte soort)

Mogelijke besmettingsroutes van de Cytostatica:
Het aanraken van besmette oppervlakken met de onbeschermde huid;
Spatten op de onbeschermde huid of in het oog;
Huidopname wanneer de handschoen doorlaat of wanneer handschoen beschadigd is;
Het inslikken van cytostatica via de besmette handen.
Wees alert dat alle gebruikte materialen die in contact zijn geweest met de patiënt besmet zijn.

Na afloop van de operatie (als de patiënt van de kamer is)
Houd de persoonlijke beschermingsmiddelen aan
Reinig alle contactpunten met patiënt
Reinig de armschelpen
Afhankelijk van de gebruikte cytostatica met water of chloor deze bovenstaande spullen reinigen. De beeldschermen en anesthesiekast niet met chloor poetsen!
Overzichtslijst waarmee te poetsen heeft de chirurgie.
Vervang de beademingsslangen en het compact blok
Het afval dat in contact is geweest met cytostatica wordt verzameld in de septoboxen (blauwe bakken) op de kamer. Indien al het afval verzameld is, sluiten we de bakken, vermeld datum en paraaf.

Bij morsen van bloed/gazen/urine/wondvocht besmet cytostatica:
Meteen een blauw doekje op de morsplaats leggen
Omloop doet een beschermende jas aan
Paarse handschoenen aandoen
Een normaal chirurgisch masker (type IIR)
Beschermende bril (uvex spatbril geel of blauw) opzetten
Omloop poetst morsplek met doekje en bleekwater van buiten naar binnen
Evt. apotheek bellen of na werktijd de dienstdoende apotheker.
Lekkage op de huid meteen wassen met water en zeep.

Bloedafname momenten i.v.m. onderzoek:
Voor de PIPAC-studie neemt de arts-onderzoeker chirurgie op de OK op vaste momenten telkens enkele ml in een EDTA buis en enkele ml in een heparinebuis af.
Dit voor farmacokinetische analyses van oxaliplatine (heparinebuis) en analyses naar (circulerend tumor)-DNA (EDTA buis). De momenten betreffen:

T=0h, voor toediening PIPAC (patiënt onder narcose)
T=0.5h, direct na toediening PIPAC bij terugkomst op de kamer (patiënt onder narcose)
T=1h op OK/recovery (patient onder narcose/wakker)
T=2h op recovery (patiënt wakker)

14.4 PROTOCOL FOR SCRUB NURSES

Protocol
Titel*
PIPAC lap sc procedure (Pressurized IntraPeritoneal Aerosol Chemotherapy), operatiekamers.

Doelstelling*
Het betreft een palliatieve ingreep met als doel regressie van de peritoneale metastasen. Hierdoor blijven patiënten mogelijk langer symptoomvrij, met een betere kwaliteit van leven en een kleine kans op dusdanige regressie dat HIPEC mogelijk is.

Indicatie

Peritoneale metastasen die niet chirurgisch verwijderd kunnen worden.
PIPAC kan herhaald worden tot de tumor uitbreidt óf laparoscopie technisch niet meer mogelijk is.
Gewoonlijk zitten er 6 weken tussen de opeenvolgende PIPAC-behandelingen.

Contra indicaties

Darmstenose
Darmletsel peroperatief
Allergie voor oxaliplatin

Plancode(s)

-

Uitvoerenden*

- Algemeen chirurgen.
- Arts-assistenten.
- Operatie-assistenten.
- MAD-ers.

Benodigdheden*

Klaarzetten op gang

Aantal	Artikelnaam	Intern artikelnummer	Bijzonderheden
1	Mayosloop large	A8036505	
1	Universeel-set 111	A8010122	
1	Röntgengazen 10 x 20 cm	P1172030	
1	Naaldenteldoos klein	D5110100	
2	Hoes Voor Videocamera 13 X 250 Cm	T1519923	
1	Slang CO2 22mm	S7659935	
1	CO2 filter	P4972210	
1	Zuigslang 5mm	S7656209	Voor Co2 filter > pleurevac
1	Diathermie met rookafzuiging 'Shark'	T1516510	
1	Groene dop thermoskan		

Instrumentennet (navision)

Naam instrumentennet	Code instrumentennet	Bijzonderheden
M.I.C. Werkinstr	E00000008	Doeken bewaren voor post operatief inpakken instrumenten
M.I.C. Inbrenginstr	E000000199	Doeken bewaren voor post operatief inpakken instrumenten
Set rubbertjes		Doeken bewaren voor post operatief inpakken instrumenten
M.I.C. camerahouder		Doeken bewaren voor post operatief inpakken instrumenten
Biopsietang		Doeken bewaren voor post operatief inpakken instrumenten
Grondblad Noord	E000000139	Bij conversie
Maag-/darmblad	E000000222	Bij conversie
Maag-/darmset extra	E000006226	Bij conversie

Wondspr. Omni-tract VF450, Bevest.	E000001354	Bij conversie
Wondspr. Omni-tract VF450, Specula	E000001352	Bij conversie

Op kamer klaarzetten

Aantal	Artikelnaam	Intern artikelnummer	Bijzonderheden
	Chloorhexidine 0,5% in alcohol 70% magenta	14951487	huiddesinfectie
	Gedest water spoelvl 500 ml pl fl	13897306	Heet
1	NaCl		Evt, bij ontbreken ascites
1	Diathermieplaten	T1511280	
1	Patienten plaat Ultravision		
	Handschoenen team		
1	Ropivacaine 10mg/ml		
2	20 cc spuit LL		
1	Groene naald		
1	Mes No.15	R7300115	
2 of 3	Polysorb 2/0 GU-46 Nld	Q5063778	
1	Caprosyn 3/0 P-12 Nld	Q5061068	
3	Hansapor 7,2 x 5 Cm Steriel	P1884602	
1	Rode maagsonde		Evt voor aspireren ascites
1	60 cc spuit CT		Evt voor aspireren ascites
2	Blunt tip trocar		Ref: OMS-T10BTNL
4	PA potjes		
2	Rode dopjes anesthesie		
1	Vernevelaar		Capnopen/MIP, testen met NaCl voor aansluiten, eiland kast F onderin pipacbak
1	Adapter		eiland kast F onderin pipacbak
1	Endoclip II ML 10mm	R6510826	Markeren biopsie laesies, alleen eerste keer PIPAC
1	Kinderfilter		Anesthesie, kast 4E
1	Zuigslang Blauw	S7656209	Afzuig pleurevac > kinderfilter
1	Pleurevac		
1	Electrode Ultravision		
2	Steriel potjes		Cytologie ascites

5	Blauwe septic bak		
	Angiopomp		Kamer 20
	Spill kit		Hipec kar
	Disposable omloopjassen		Voor omloop/anesthesie
	Mondmasker FFP3	A8511520	Hipec kar
	Brillen uvex astrospec	BA:165207	Hipec kar
4	Blauwe onderleggers		Hipec kar
	Disposable doekjes voor morsen		Hipec kar
	Instrumentenkar		Voor sets
3	Kaarten Cytostatica		Op kamer en kar hangen
	Optimum +filter + connector		

Algemene opmerkingen
- Apparatuur op werking controleren
- Alle materialen, protheses controleren op aanwezigheid.
- Volledige OK-team aanwezig dan Time Out Procedure
- Handhygiene niet snijdend team

Na deze ingreep is de kamer 'besmet', hierna alleen nog andere PIPAC operaties op deze kamer
Altijd voorbereid zijn op conversie

Werkwijze*
Voorbereiding
Algehele voorbereiding voor PIPAC procedure:
Huishoudelijke dienst inlichten.
Schoonmaken kamer na de procedure.
CS informeren, bellen wanneer er definitief verneveld wordt.
Kar voor instrumenten VOOR de OK plaatsen met bordje "Cytostatica" en 1 Septobox
Controle HIPEC omloop kar.
High pressure pomp (injector) op OK
Controle schermen controlehokje.
Ultravision

Apotheek informeren : is de cytostatica bereid? tel:8382 of 114790

Waarschuwingsbordjes op deur hangen.

Aansluiten filtersysteem (pleurevac tot optimum)
trocac > CO2filter > 5mm zuigslang waar blauw stukje afgeknipt is > pleurevac met waterslot tot 20mm/hg gevuld > 5mm zuigslang > kinderfilter anesthesie > rookafzuigfilter > connector CO2 verwarmingsslang (adapter, with tube, insufflation 031822-01) > optimum

Aansluiten filtersysteem (pleurevac tot muur):
trocar > Co2 filter > 5mm zuigslang waar blauw stukje afgeknipt is > pleurevac met waterslot tot 20 mm/hg gevuld > 5mm zuigslang > kinderfilter anesthesie > rookafzuigfilter

Voorbereiding patiënt

Ligging

Rugligging.
Blauwe onderlegger onder patiënt leggen i.v.m. morsen cytostatica (denk aan vouwen ivm doorliggen).
Beide armen uit.
Diathermieplaat + connecteren met Ultravision op bovenbenen.
3 blauwe onderleggers op de grond om de tafel
halve blauw onderleggen onder injector

Opstelling team en apparaten

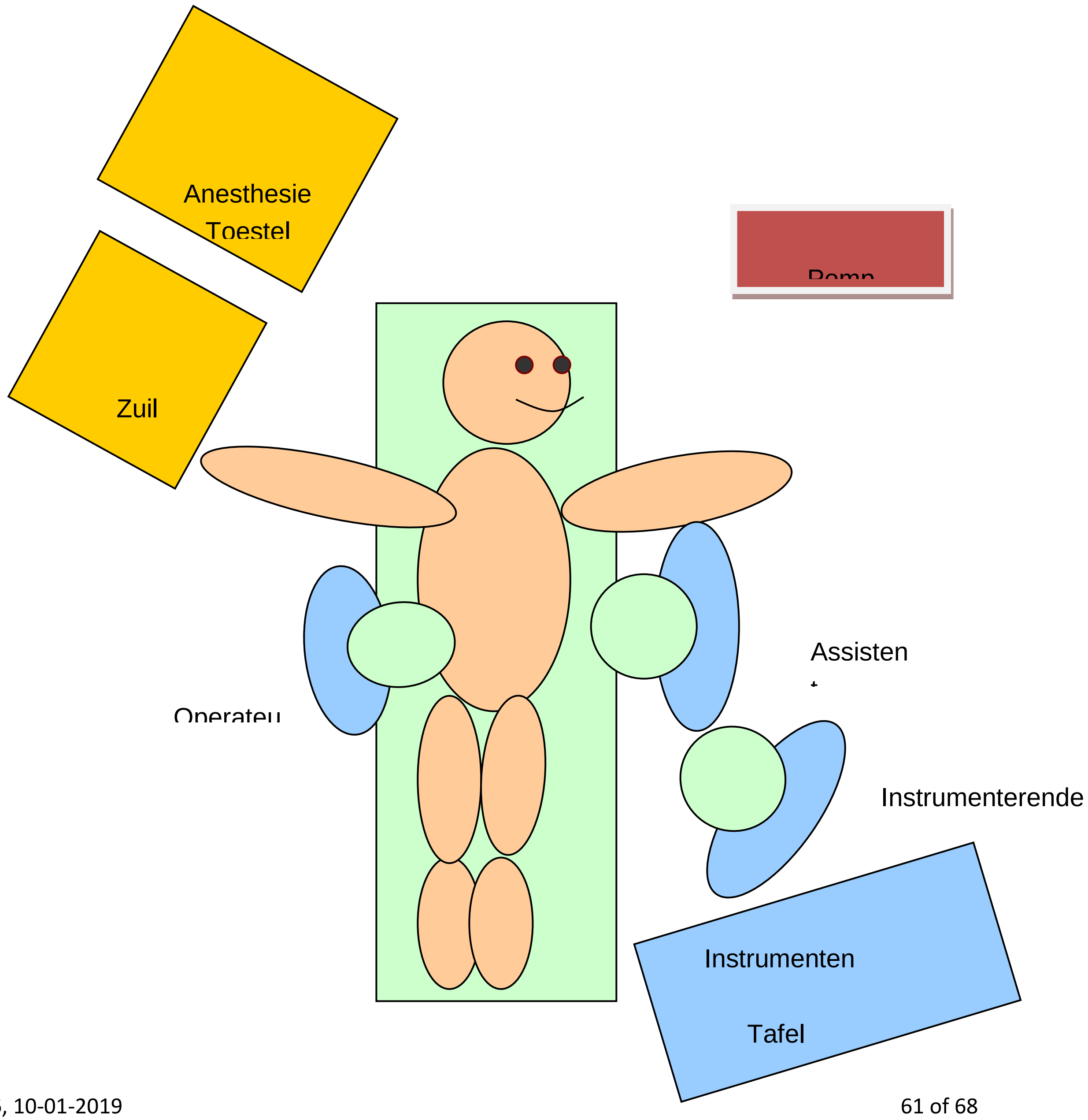

Voorbereiding snijdend team

Handen desinfectie volgens protocol.
Steriel aankleden snijdend team volgens protocol.
Chirurg start OK in HIPEC tenue. (Hipec jas, dubbele handschoenen, FFP3 masker, spatbril)

Operatieprocedure/verloop

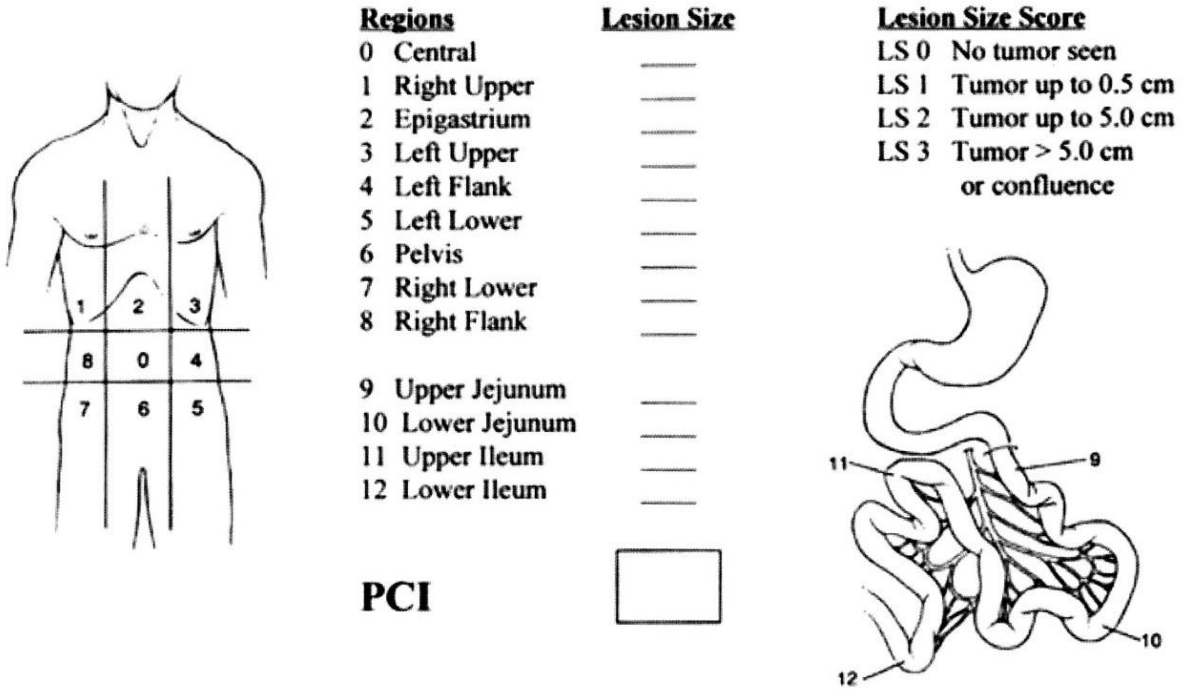

Biopten worden genomen op vier kwadranten van het peritoneum, deze worden weggestuurd voor 'vriescoupe' o.v.v. CRC-PIPAC studie
Ascites wordt opgezogen en ingestuurd voor cytologie (2x steriel potje) o.v.v. CRC-PIPAC studie. Bij geen ascites, enkele cc NaCl inspuiten, opzuigen, **direct insturen** voor cytologie.
Test van gaslekkage. (zet evt de insufflator op 0) Evt extra hechting of doekenklem plaatsen om gaslekkage te stoppen.
Nu alle specifieke PIPAC spullen uitpakken.
Vernevelaar testen met 20cc spuit met NaCl ivm mogelijk lekken.
De vernevelaar inpakken in camerahoes (karton na inpakken eraf halen) ivm voorkomen evt lekken + 5 mm rubbertje om de vernevelaar plaatsen.
Op de cameratrocar komt de CO2 filter + zuigslang (afzuig) welke geconnecteerd wordt aan de pleurevac, rolletje van CO2 filter rolletje **dichtzetten!** Op de uitgang van ballon rood anesthesie dopje plaatsen.
De camera houdt de vernevelaar in beeld en mag in geen geval de ingewanden raken ivm de hitte van de lichtbron. De camera fixeren in de PIPAC camerahouder.
Door de andere trocar komt de vernevelaar. De tip van de vernevelaar moet enkele mm uit de trocar steken. Deze fixeer je met een rubbertje van de set. Op de trocar komt net zoals bij de andere trocar een rood dopje van de uitgang van de ballon.

Spuit met tip naar boven in pomp plaatsen. Dopje er voorzichtig afdraaien. High pressure line van de vernevelaar aansluiten op de spuit. Camerahoes fixeren met tape aan spuit. Positioneren spuit en boven septobox hangen. Septobox staat op blauwe onderlegger.
Alles wat je moet weten van de pomp:
Nummer 13 is het pipac programma. Na deze aan te hebben geklikt druk op air check, daarna druk op accept bolus > Start OK Hierna is de pomp gereed.
Wanneer alles is aangesloten: Checklist
Alle zakken dichtknopen maar wel op de kamer laten.
Nu alleen nog maar septo bakken gebruiken.
Iedereen naar buiten
Hipec bordjes ophangen.
Start injectie met voetpedaal/handbediening buiten OK, de knop moet 5 minuten ingedrukt blijven totdat de spuit met cytostatica leeg is.
Één persoon blijft steriel op buitengang ivm mogelijke calamiteit.
Verneveling start, dit duurt in totaal 30 minuten.
Na 5 minuten één persoon terug naar binnen om ultra vision aan te zetten, zodat aerosol neerslaat op het weefsel van de patiënt.
Na 30 minuten gaat de chirurg + omloop (met HIPEC bescherming) als eerste terug naar binnen
Omloop zet CO2 uit in monitorkamer
Chirurg verwijdert electrode ultra vision (opening afdekken) en zet de afzuig open. Hierna volgt de rest van het team in HIPEC tenue (blauwe overjas, onsteriele handschoenen, FFP3 masker, spatbril).
Verwijderen trocars
Belangrijk voor de omloop: Bij verwijderen spuit uit pomp, eerst slang van spuit loskoppelen voor de stamper naar beneden te halen. (dit om te voorkomen dat het systeem vacuüm zuigt)
Sluiten fascie en huid
Injectie wondjes met ropivacaine.

Bij morsen van de Cytostatica
Lees crashcard cytostatica en omgaan met cytostatica
Meteen een absorberend doekje op de morsplaats leggen.
Handschoenen aan.
Masker FFP3 met ventiel.
Beschermende bril.
Omloop poetst morsplek van buiten naar binnen.
Bij oxaliplatine met water en zeep.
Bij vragen eventueel apotheek bellen of na werktijd dienstdoende apotheker.
Lekkage **<u>OP</u>** de huid, meteen wassen met water en zeep.

Opruimen instrumentarium
Instrumentenkar op de kamer zetten.
Alle instrumentennetten inpakken in de originele verpakking, instrumennet van MIC werkinstrumenten uit elkaar halen.

Brillen die niet besmet zijn, per 3, in plastic zakje doen voor reiniging, en in de kar met instrumenten meegeven naar de CS.
1x grote septic box op de kar zetten! Als ook het bordje cytostatica aan kardeur plaatsen.
Kar sluiten en kar naar CS brengen en aanmelden.
Dus je weegt de instrumenten **NIET**. Let op tellen!
Lampendoppen ook inpakken.

Opruimen operatiekamer
Bloedspetters op vloer en apparatuur meteen verwijderen, maar wat je nog ziet schoonmaken met disposable doekjes, (oxaliplatine met water en zeep)
Alle afval in de septic boxen doen en afsluiten, beschouw al het afval als gecontamineerd.
Instrumententafels laten staan.
Huishoudelijke dienst bellen
Filters verwijderen in de septic box doen
Omloopkar / Oesophaguskar / Hipec omloopkar kan buiten op de gang.

Wat te doen bij calamiteit tijdens vernevelen
Bij de eerste 5 min, stopzetten van de vernevelaar. Na die eerste 5 minuten kan deze stap over geslagen worden.
Chirurg in beschermende kleding naar binnen om CO2 stop te zetten, ultravision activeren en actieve afzuig aanzetten.
Indien mogelijk, qua veiligheid patiënt, hierna 30 sec wachten totdat de ultravision alle ionen heeft doen neerslaan.
Daarna kan iedereen in beschermende kleding naar binnen.

Administratie en post-operatief
Bijlagen
Handen desinfectie (hyperlink).
Steriel aankleden snijdend team (hyperlink).
Preparaten naar PAMM (hyperlink)
Schoonmaken operatiekamer na HIPEC procedure (hyperlink)
Time Out Procedure
Crashcard cytostatica en omgaan met cytostatica

Literatuur

Auteur*
Kim Blox & Marieke ten Hoopen, operatie assistent
Beoordelaars*
Dr de Hingh, algemeen chirurg
Suzanne Jacobs, kwaliteitsadviseur
Hygiëne en infectiepreventie
Petra Kerstens, operatie assistent

Autorisator*
Einde document

15. FIGURES & TABLES

Figure 1. Flow chart of the CRC-PIPAC study.

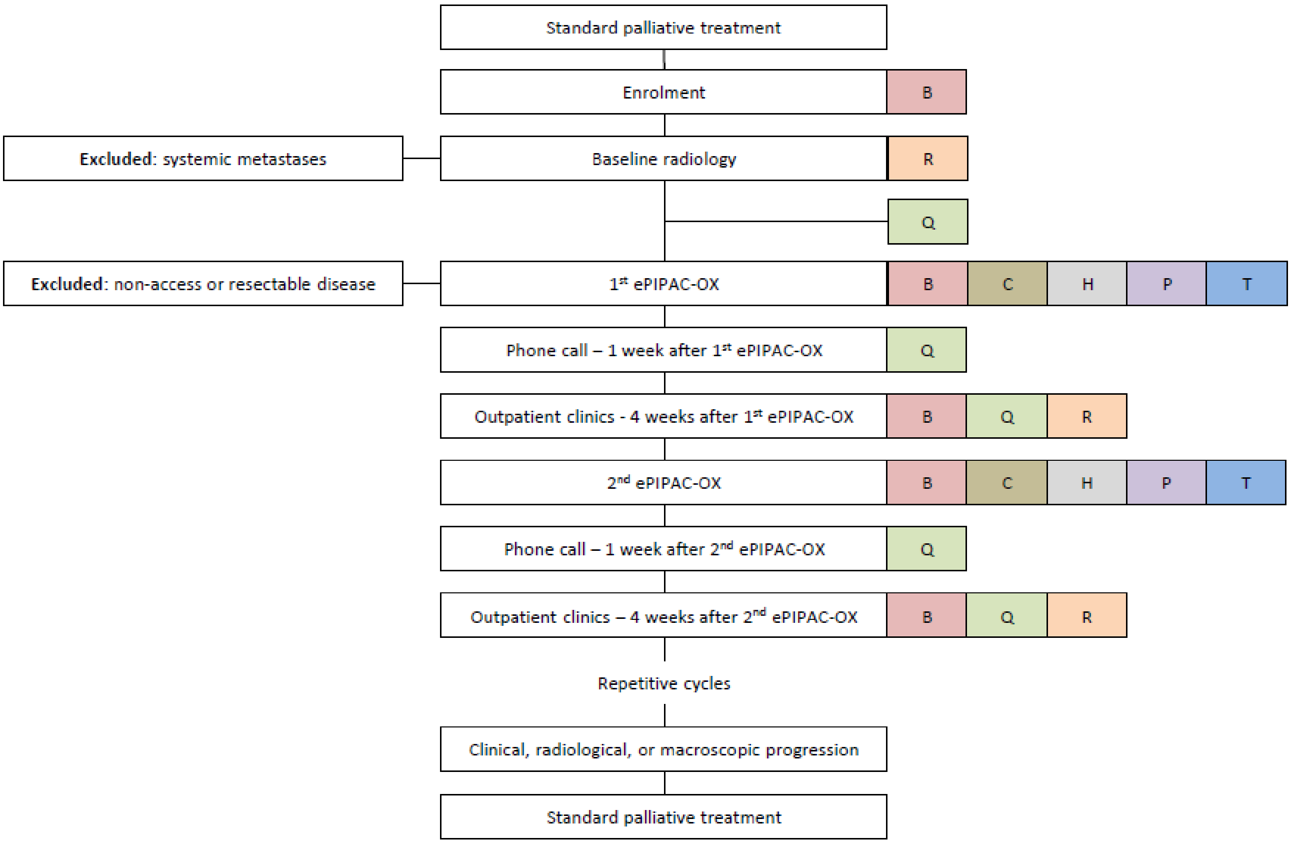

Figure 1. *B* bloods (organ toxicity, tumour markers); *C* cytology (ascites or peritoneal washing with saline); *ePIPAC-OX* electrostatic pressurised intraperitoneal aerosol chemotherapy with oxaliplatin; *H* histopathology (peritoneal biopsies); *P* pharmacokinetics (blood, urine, ascites, PM, normal peritoneum); *Q* questionnaires (quality of life, costs); *R* radiology (thoracoabdominal CT, diffusion-weighted MRI); *T* translational research (blood, ascites, PM).

Table 1. Schedule of enrolment, interventions, and assessments.

	STUDY PERIOD								
	Enrolment/allocation	Post-enrolment							
	Outpatient clinics	*Baseline radiology*	*1st ePIPAC-OX*	*1 week after 1st ePIPAC-OX*	*4 weeks after 1st ePIPAC-OX*	*2nd ePIPAC-OX*	*1 week after 2nd ePIPAC-OX*	*4 weeks after 2nd ePIPAC-OX*	*Repetitive cycles*
ENROLMENT/ALLOCATION									
Eligibility screen	X								
Informed consent	X								
INTERVENTIONS									
ePIPAC-OX			X			X			X
Blood (organ functions, tumour markers)	X		X[A]		X	X[A]		X	X
Pharmacokinetics (blood, urine, ascites, PM, normal peritoneum)[B]			X			X			X
Translational research (blood, ascites, PM)			X[C]			X[C]			X
Thoracoabdominal computed tomography		X			X			X	X
Diffusion-weighted magnetic resonance imaging		X			X			X	X
Cytology (ascites or peritoneal washing)			X			X			X
Histopathology (peritoneal biopsies)			X			X			X
Questionnaires: quality of life		X		X	X		X	X	X
Questionnaires: costs		X[D]		X[E]	X		X[D]	X	X
ASSESSMENTS									
Baseline characteristics	X	X	X						
Toxicity			X	X	X	X	X	X	X
Environmental safety of ePIPAC-OX			X[F]						
Procedure-related characteristics			X			X			X
Number of procedures in each patient, reasons for discontinuation			X	X	X	X	X	X	X
Organ-specific toxicity			X		X	X		X	X
Postoperative complications			X	X	X	X	X	X	X
Hospital stay			X			X			X
Readmissions				X	X		X	X	X
Clinical evaluation			X	X	X	X	X	X	X
Radiological tumour response		X			X			X	X
Histopathological tumour response			X			X			X
Cytological tumour response			X			X			X
Biochemical tumour response			X		X	X		X	X
Quality of life		X		X	X		X	X	X
Costs		X		X	X		X	X	X
Progression-free survival			X	X	X	X	X	X	X
Overall survival			X	X	X	X	x	X	X

ePIPAC-OX electrostatic pressurised intraperitioneal aerosol chemotherapy with oxaliplatin; *PM* peritoneal metastases; [A]drawn on each postoperative day; [B]blood is collected before ePIPAC-OX and 5, 10, 20, 30, 60, 120, 240, 360, and 1080 minutes after oxaliplatin injection during/after the first three procedures, urine is collected before ePIPAC-OX and on postoperative days 1, 3, 5, and 7, ascites/PM/normal peritoneum are collected during ePIPAC-OX, directly after oxaliplatin injection; [C]blood is drawn before each ePIPAC-OX; [D]PCQ and MCQ; [E]PCQ; [F]only during the first three procedures in the study.
